# Time-Dependent Changes in Depressive Symptoms Among Control Participants in Digital-Based Psychological Intervention Studies: Meta-analysis of Randomized Controlled Trials

**DOI:** 10.2196/39029

**Published:** 2023-04-12

**Authors:** Alan CY Tong, Florence SY Ho, Owen HH Chu, Winnie WS Mak

**Affiliations:** 1 Department of Psychology The Chinese University of Hong Kong New Territories Hong Kong

**Keywords:** digital-based psychological intervention, control groups, meta-analysis, depression, depressive symptoms, mobile phone

## Abstract

**Background:**

Digital-based psychological interventions (DPIs) have been shown to be efficacious in many randomized controlled trials (RCTs) in dealing with depression in adults. However, the effects of control comparators in these DPI studies have been largely overlooked, and they may vary in their effects on depression management.

**Objective:**

This meta-analytical study aimed to provide a quantitative estimate of the within-subject effects of control groups across different time intervals and explore the moderating effects of control types and symptom severity at baseline.

**Methods:**

A systematic literature search was conducted in late September 2021 on selected electronic databases: PubMed; ProQuest; Web of Science; and the Ovid system with MEDLINE, PsycINFO, and Embase. The control conditions in 107 RCTs with a total of 11,803 adults with depressive symptoms were included in the meta-analysis, and effect sizes (Hedges *g*) were calculated using the standardized mean difference approach. Study quality was assessed using the Cochrane risk-of-bias tool for randomized trials version 2.

**Results:**

The control conditions collectively yielded small to moderate effects in reducing depressive symptoms within 8 weeks since the baseline assessment (*g=*−0.358, 95% CI −0.434 to −0.281). The effects grew to moderate within 9 to 24 weeks (*g=*−0.549, 95% CI −0.638 to −0.460) and peaked at *g=*−0.810 (95% CI −0.950 to −0.670) between 25 and 48 weeks. The effects were maintained at moderate to large ranges (*g=*−0.769, 95% CI −1.041 to −0.498) beyond 48 weeks. The magnitude of the reduction differed across the types of control and severity of symptoms. Care as usual was the most powerful condition of all and produced a large effect (*g*=−0.950, 95% CI −1.161 to −0.739) in the medium term. The findings showed that waitlist controls also produced a significant symptomatic reduction in the short term (*g=*−0.291, 95% CI −0.478 to −0.104), refuting the previous suspicion of a nocebo effect. In addition, a large effect on depressive symptom reduction in the long term (*g*=−1.091, 95% CI −1.210 to −0.972) was noted among participants with severe levels of depressive symptoms at baseline.

**Conclusions:**

This study provided evidence that depressive symptoms generally reduced over time among control conditions in research trials of DPIs. Given that different control conditions produce variable and significant levels of symptomatic reduction, future intervention trials must adopt an RCT design and should consider the contents of control treatments when investigating the efficacy of DPIs. The results of waitlist controls confirmed previous findings of spontaneous recovery among people with mild to moderate depressive symptoms in face-to-face studies. Researchers may adopt watchful waiting as participants wait for the availability of digital-based psychological services.

## Introduction

### Digital-Based Psychological Interventions for Depression

Depressive disorders are among the most frequently occurring mental disorders, with a prevalence rate of 10.7% within a 12-month period [1]. They affect >280 million people worldwide [2] and are ranked fourth in terms of disease burden [3]. At the individual level, they have adverse effects on one’s cognitive functioning, quality of life, mortality, and other health outcomes [4-6]. At the macro level, they have tremendous economic costs worldwide [7,8].

Psychological interventions, especially cognitive behavioral therapy (CBT), have demonstrated to be efficacious in treating depressive disorders [9-12]. They are not only as efficacious as antidepressant medications [13], but they are also preferred by people with depression [14]. Despite its demonstrated efficacy, the uptake of psychotherapy has been poor, with only 13.8% of those who experience mood disorders receiving psychological interventions [15]. With the emergence of digital-based psychological interventions (DPIs), the uptake of evidence-based psychotherapy may be expedited through access over the internet.

A psychological intervention is considered digital when technology is used in its delivery [16]. Although varying in forms, DPIs typically use a software program, website, or app to disseminate therapeutic contents displayed in texts, audios, or videos [17]. Although most modern DPIs run on the internet, offline interventions may also be considered digital whenever technology is involved, for example, a therapy program installed on a web-free computer or that runs on a CD-ROM [18]. Nonetheless, some DPIs would also come with human support, for instance, having clinicians to review homework assignments and provide feedback on users’ progress [19].

DPIs have overcome major help-seeking barriers by reducing time and costs [20] and mitigating stigma through anonymity and privacy [21]. DPIs’ effectiveness in treating common mental health conditions was found to be comparable with that of face-to-face psychological interventions [22,23]. Previous meta-analytic studies [24-27] have demonstrated their efficacy in alleviating depressive symptoms among adults when compared with a control group, with between-group effect sizes ranging from *g=*−0.41 to *g=*−0.90.

### The Overlooked Value of Control Groups

Changes in depression during treatment can be attributed to at least 5 distinct sources of effects [28]. These include the treatment-specific effect, which is the net effectiveness of a treatment; the nonspecific treatment effects such as attention, support, and expectations of being treated; spontaneous remission; regression to the mean; and other treatment-unrelated factors. Unlike pharmacological trials, where blinding is relatively easy to carry out, participants in psychological intervention trials know immediately the results of assignment upon allocation. Any changes in the outcome can be regarded as a blend of true treatment effects and an array of other non–treatment-specific effects. In other words, a proportion of changes experienced by the clients or users during the therapy process may come from sources other than the designated treatment.

Ideally, randomized controlled trials (RCTs) that use adequate control conditions should have controlled for these nontreatment effects [29]. The established evidence we have today is largely generated by RCTs and has been consolidated in meta-analyses that focused on between-group effects comparing an intervention with a control group. In a recent meta-analysis, Moshe et al [27] found that the types of control conditions significantly moderated the effect sizes of DPIs, implying a therapeutic potential in these control conditions.

### Previous Reviews on Control Groups

Mohr et al [30] conducted a comprehensive review of the control conditions in RCTs examining the efficacy of psychological treatments for depression. They found that the choice of control condition can have a very large impact on the outcome of a study. In general, studies that used a waitlist or treatment-as-usual control produced the largest between-group effect favoring the examined treatment, whereas those with no-treatment or pill or psychological placebo controls found moderate effects in active treatment conditions, and those with active controls found the least effects in active treatment conditions.

Cuijpers et al [31] attempted to estimate the effects of nontreatment factors in a meta-analysis by investigating the effects of face-to-face nondirective supportive therapy. They found that nearly one-third of the observed changes in depression could be attributed to spontaneous remission and nearly half of the contribution could be accounted for by nonspecific factors. Subsequently, in their meta-analysis investigating remission in people receiving face-to-face psychological treatment, they noted an 18% reduction in depression (Beck Depression Inventory-II) scores among people in the control conditions [32]. More recently, in light of the high heterogeneity of the commonly used care-as-usual (CAU) controls, Cuijpers et al [33] investigated the different categories of CAU and found that the effects of psychotherapies did not differ significantly across CAU variants.

Another meta-analytic study conducted by Whiteford et al [34] examined the probability of remission from untreated depression across 19 studies and reported a high rate of spontaneous remission (53% within 12 months) among participants in waitlists and primary care settings. It was noteworthy that Furukawa et al [35] compared waitlist, no-treatment, and psychological placebo controls with face-to-face CBT and also found that different control conditions lead to substantial but different treatment effect estimates. The waitlist was regarded by the authors as a “nocebo” condition given its inferiority as compared with the no-treatment controls, in which the participants do not expect to receive any active treatment after the study period is over.

### Rationale and Research Questions

The aforementioned findings indicate a potential moderating effect of the control treatment on the reported effect sizes of face-to-face psychotherapies. Given that investigations have focused primarily on face-to-face psychotherapies, how control conditions perform in general in RCTs of DPIs has not been systematically investigated. In the past decade, the use of DPIs for health care has become a burgeoning area of investigation. A massive demand on the use of DPIs has been particularly evident since the COVID-19 pandemic lockdowns, when face-to-face services were stalled [36].

Participants who joined face-to-face psychotherapies might have had different demographic profiles and preferences from those who were willing to receive DPIs. The latest research on the digital divide has shown that digital use is greatly influenced by sociodemographic factors and lifestyle differences [37]. Thus, participants in DPI studies may represent a heterogeneous subgroup of individuals different from those who opt for face-to-face therapies. Updated insights into the characteristics of these individuals are warranted. Nevertheless, an in-depth understanding of how control conditions in RCTs of DPIs fare is necessary for researchers, practitioners, and consumers to evaluate the current evidence on DPIs.

This systematic review and meta-analysis aimed to investigate the possible effects of common control comparators in studies on DPIs for depression among adults. The primary objective of this meta-analytic review was to provide answers to the following research questions: (1) What are the sizes of the within-subject effects of the control conditions over time? (2) How do different types of control conditions differ in terms of time effects? and (3) How does baseline severity affect the change trajectory in control condition participants over time?

## Methods

This study adhered to the PRISMA-P (Preferred Reporting Items for Systematic Review and Meta-Analysis Protocols) [38] and has been registered in PROSPERO (CRD42021261620).

### Inclusion and Exclusion Criteria

This meta-analysis included studies that fulfilled the following criteria pertaining to *participants*, *intervention,* and *publication type*.

#### Participants

We included studies on adults with any severity of symptoms along the depressive disorder spectrum, ranging from a formal diagnosis of major depressive disorder or persistent depressive disorder to the presence of depressive episodes (major depressive episode) to elevated depressive symptoms. The exclusion criteria included being adolescents or children (aged <18 years) or older adults (aged >65 years) and having comorbid general medical conditions (eg, cancer and diabetes) and other psychiatric disorders (eg, posttraumatic stress disorder, bipolar disorder, and psychotic disorder) except for generalized anxiety disorder as it has been found to be highly comorbid with depression in the real world.

#### Intervention

We included RCTs in which the efficacy of a DPI treatment was compared with that of a control group. The operational definition of a DPI is a nonpharmacological therapeutic procedure that uses at least one therapeutic approach (eg, CBT, psychodynamic therapy, or positive psychology). The intervention must be non–face to face, delivered either through the internet using any device, including a computer, tablet, or smartphone, or offline via a computerized platform (eg, using a CD-ROM). Furthermore, we excluded studies that targeted changing participants’ physical exercise, diet, or lifestyle purposefully. Studies solely with telephone support from a health care professional were also excluded as we did not consider the telephone as a form of technology, and therefore, it did not qualify for the operational definition of DPI in this study.

#### Publication Type

We included only peer-reviewed RCTs published in an English academic journal, excluding qualitative studies, dissertations, protocols, and review papers. For controlled trials comparing 2 active interventions, we checked whether the control group delivered the same intervention but in different formats (eg, internet-based CBT with vs without support). We excluded these studies if a stand-alone control group was not present. For nonprimary studies (eg, moderator analysis and economic analysis), we checked whether a record of change in depressive symptoms over time was available.

### Study Identification

A systematic literature search was carried out on September 28, 2021, on selected electronic databases: PubMed; ProQuest; Web of Science; and the Ovid system with MEDLINE, PsycINFO, and Embase. Details of the search strategy are presented in Multimedia Appendix 1. This search strategy resulted in a total of 9249 titles and abstracts across the different databases. All search results were entered into Zotero (Corporation for Digital Scholarship), a reference manager for review and removal of duplicate citations [39]. After the removal of duplicates, a total of 49.41% (4570/9249) of the records were retained.

A total of 3 researchers (AT, FH, and OC) then screened the titles and abstracts to eliminate records that obviously did not meet the inclusion criteria (eg, studies with children or older persons, not related to depression, and not on DPIs). Excellent interrater reliability was established (intraclass correlation coefficient=0.964) between the 3 reviewers based on the first 2.19% (100/4570) of screened articles.

**Figure 1:**
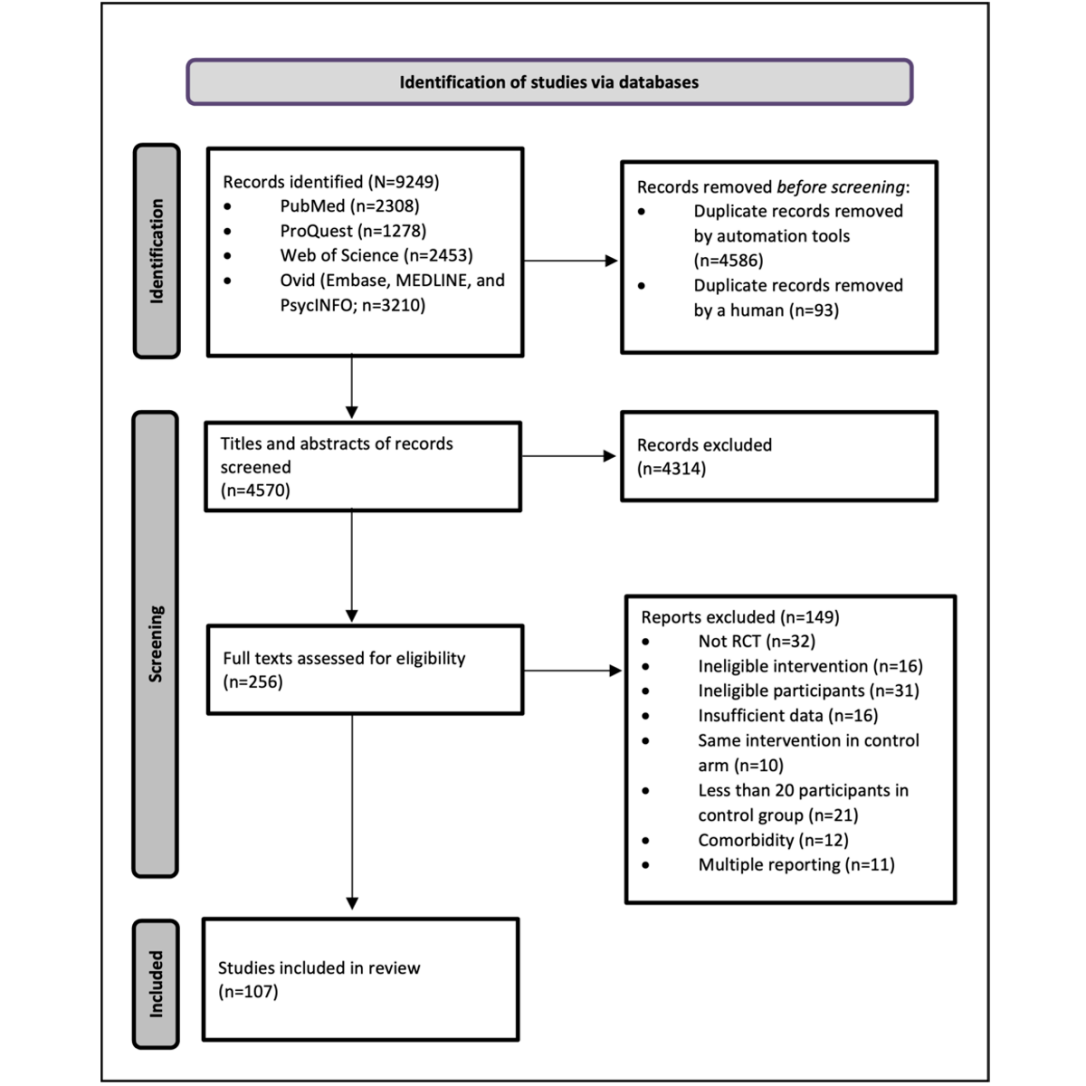
Flowchart of the study selection process. RCT: randomized controlled trial.

A total of 256 records that potentially met the inclusion criteria were identified, and the reviewers then read the full texts of these records to confirm their eligibility. Eventually, 41.8% (107/256) of these studies were left for data extraction. Figure 1 presents the PRISMA (Preferred Reporting Items for Systematic Reviews and Meta-Analyses) flowchart of the selection process.

### Data Extraction and Coding

Participant characteristics in each study, including age, the percentage of female participants, and tertiary education, were recorded. The percentage of participants taking antidepressant medications at the time of the study and previous exposure to psychotherapy were also extracted.

In addition to the therapeutic approach (eg, CBT, psychodynamic therapy, or positive psychology) of the comparator intervention (in the intervention group), information related to the control group, including types (see detailed descriptions in the *Results* section), a description of the intervention received by the control participants, and the mode of delivery, was also extracted. In the event of studies having more than one control group (eg, a waitlist and an active control), we extracted both conditions as separate data as the results for these subsamples were considered to reflect the outcomes of 2 studies. The time passed (in weeks) since the baseline assessment and the retention rate among the controlled participants at each assessment time point were also recorded.

The primary outcome of interest was the within-subject change in depressive symptoms over time. Wherever available, the observed means and SDs of depressive symptoms at baseline, postintervention measurement, and follow-ups were extracted. SEs were converted into SDs by multiplying the square root of the sample size. When the aforementioned information was not available, we checked whether effect sizes with CIs and mean changes with SDs were available. In cases where more than one depressive symptom measure was used, all of them were extracted and aggregated in the meta-analysis.

### Quality Assessment

Study quality was assessed using the Cochrane risk-of-bias tool for randomized trials version 2 (RoB 2) [40]. The RoB 2 is a structured tool that assesses possible sources of bias in a trial across five different domains: (1) the randomization process, (2) deviation from the intended interventions, (3) missing outcome data, (4) measurement of the outcomes, and (5) selection of the reported results. A high-quality study should be at low risk in all 5 domains. In total, 2 reviewers (FH and OC) independently assessed the studies based on the RoB 2, and a third reviewer (AT) made the final decision whenever there was disagreement between the reviewers.

### Data Synthesis and Meta-analysis

Meta-analyses were performed using Comprehensive Meta-Analysis (version 3; Biostat, Inc) [41] to determine the mean differences in depressive symptoms across different time durations (in weeks) since baseline. The duration was divided into four intervals: (1) immediate (≤8 weeks), (2) short term (9-24 weeks), (3) medium term (25-48 weeks), and (4) long term (>48 weeks). These time cutoffs were chosen based on the distribution of the assessment time points throughout the selected studies. In cases where more than one assessment time point fell within the same interval (eg, 18 and 24 weeks), we used the one closer to the far end of that interval (ie, 24 weeks).

The correlation between pre-post scores on the outcome measures was assumed to be *r*=0.59 with reference to Balk et al [42]. Effect sizes (Hedges *g*) were calculated using the standardized mean difference (SMD) approach. The interpretation of effect size follows Cohen [43], where 0.2 represents a small effect, 0.5 represents a moderate effect, and 0.8 represents a large effect. The extent of heterogeneity between the studies was assessed using the *I*^2^ statistic, where a value of ≥75% represents significant heterogeneity. Random-effects models were calculated assuming significant heterogeneity [44].

Publication bias was examined using funnel plot inspection and Egger regression [45]. Symmetry of the funnel plot and a nonsignificant Egger regression indicated an absence of publication bias. Whenever publication bias was detected, the trim-and-fill procedure by Duval and Tweedie [46] was used to obtain a sensitivity estimate of the effect size.

Apart from the differences in effect sizes at various time intervals, subgroup analyses were performed to explore the differential impacts of control group types on severity of depressive symptoms in effect sizes. Only subgroups that comprised ≥2 studies were interpreted to ensure the stability of the effect size estimates.

Depressive symptom severity was categorized into 3 levels—mild, moderate, and severe—based on the proposed cutoffs provided in the respective publications (see Multimedia Appendix 1 for the cutoffs). Finally, we also explored the meta-regression effects of age, sex ratio, and higher education as an exploratory analysis in explaining the heterogeneity across the studies.

## Results

### Overview of the Included Studies

A total of 107 peer-reviewed RCTs meeting the inclusion criteria with an aggregation of 11,803 control participants were included in this meta-analysis. The studies were conducted between 2003 and 2021. The years with the most publications were 2020 and 2021, each with 13.1% (14/107) of the studies included. More than half of them (60/107, 56.1%) were conducted in Europe (eg, the Netherlands, Germany, and Sweden) and the United Kingdom. Australia (17/107, 15.9%) and the United States (18/107, 16.8%) also contributed substantially. Other regions included China, Singapore, Japan, Nigeria, Oman, and Colombia. Most of these studies recruited participants from the community (52/107, 48.6%) and in clinics (36/107, 33.6%).

The reported mean age of the participants was 38.48 (SD 7.69) years, and the mean percentage of female participants was 71.54% (SD 15.38%). Approximately half of the participants (mean 54.1%, SD 20.66%) attained higher education, as defined by having finished college or university, and were either married or living with partners (mean 52.26%, SD 19.98%), and 68.19% (SD 19.12%) were employed (including being a student) at the time of the study. Although not reported in many studies, the mean percentage of participants taking antidepressant medication was 44.86% (SD 23.59%), and 51.55% (SD 22.41%) had experience receiving psychotherapy before the study, as reported in 52.3% (56/107) and 38.3% (41/107) of the studies, respectively. The most frequently used assessments of depressive symptoms were the Patient Health Questionnaire-9 (43/107, 40.2%) and the Beck Depression Inventory-II (40/107, 37.4%). Key features of the 107 included studies are summarized in Table 1.

**Table 1:** Summary of the included studies (N=107).

Study, year	Country	Setting	Selection criteria (depression)	Comparator intervention	Control type	Scale	Time interval
Al-Alawi et al [47], 2021	Oman	Community	PHQ-9^a^ ≥12	iCBT^b^+iACT^c^	Information	PHQ-9	I^d^
Andersson et al [48], 2005	Sweden	Community	MDD^e^ (CIDI^f^); MADRS-S^g^=15-30	iCBT	Active	BDI^h^ and MADRS-S	S^i^ and M^j^
Beevers et al [49], 2017	United States	Community	QIDS^k^ >10	iCBT	CAU^l^	QIDS-SR^m^ and HDRS^n^	I
Berger et al [50], 2018	Switzerland	Clinic	BDI-II >13	iCBT	Active	BDI-II^o^	S and M
Birney et al [51], 2016	United States	Workplace	PHQ-9=10-19	iCBT (mindfulness-enhanced)	Information	PHQ-9	S
Bohlmeijer et al [52], 2021	The Netherlands	Community	CES-D^p^ <34	Gratitude intervention	Active	CES-D	I, S, and M
Bohlmeijer et al [52], 2021	The Netherlands	Community	CES-D <34	Gratitude intervention	WL^q^	CES-D	I and S
Boschloo et al [53], 2019	The Netherlands	Multiple	PHQ-9=5-14	iCBT	CAU	PHQ-9	S
Braun et al [54], 2021	Germany	Workplace	PHQ-9 ≥5	iCBT	Information	QIDS-SR	S
Browning et al [55], 2012	United Kingdom	Community	Recurrent depression (SCID^r^)	Attentional bias modification	Sham	BDI-II and HDRS	I
Buntrock et al [56], 2015	Germany	Workplace	CES-D ≥16	iCBT	Information	CES-D	I and M
Calkins et al [57], 2015	United States	University	BDI-II score=17-34	CCT^s^	Sham	BDI-II	I
Choi et al [58], 2012	Australia	Community	MDD (DSM-IV^t^)	iCBT	WL	BDI-II and PHQ-9	S
Clarke et al [59], 2005	United States	Community	MDD	iCT^u^	Information	CES-D	I, S, and M
Clarke et al [60], 2009	United States	Community	MDD	iCBT	Information	PHQ-8^v^	I, S, M, and L^w^
Dainer-Best et al [61], 2018	United States	Community	CES-D >13	PSRT^x^	Sham	CES-D	I
Day et al [62], 2013	Canada	University	DASS-21-D^y^ >10	iCBT	WL	DASS-21-D	I
De Graaf et al [63], 2011	The Netherlands	Community	BDI-II >16	iCBT; iCBT+CAU	CAU	BDI-II	I, S, and M
Eriksson et al [64], 2017	Sweden	Clinic	MDD (MINI^z^); MADRS-S <35	iCBT; iCBT+CAU	CAU	BDI-II	S and M
Flygare et al [65], 2020	Sweden	Clinic	MADRS-S=15-30	iCBT	Sham	BDI-II and MADRS-S	I, M, and L
Fonseca et al [66], 2020	Portugal	Clinic	PDPI-R^aa^ ≥5.5; EPDS^ab^ ≥10	iCBT	CAU	EPDS	I
Geraedts et al [67], 2014	The Netherlands	Workplace	CES-D >16	iPST^ac^+iCT	CAU	CES-D	I, M, and L
Gilbody et al [68], 2015	United Kingdom	Clinic	PHQ-9 ≥10	iCBT	CAU	PHQ-9	S, M, and L
Gili et al [69], 2020	Spain	Clinic	PHQ-9=5-14	Healthy lifestyle program, mindfulness program, and positive affect promotion program	CAU	PHQ-9	I and M
Hallford et al [70], 2021	Australia	Community	PHQ-9 ≥10; MDE^ad^	Memory Specificity Training	WL	PHQ-9	I and S
Hallgren et al [71], 2016	Sweden	Clinic	PHQ-9 ≥9	iCBT	CAU	MADRS-S	S and L
Hange et al [72], 2017	Sweden	Clinic	MADRS-S <35; MDD (MINI)	iCBT	CAU	MADRS-S	S, M, and L
Harrer et al [73], 2021	Germany	University	CES-D ≥16	Stress intervention	Information	CES-D	I and S
Hatcher et al [74], 2018	Canada	Clinic	MDD; dysthymia	iCBT+PST^ae^	Information	PHQ-9	I and S
Heim et al [75], 2021	Switzerland	Community	PHQ-9 ≥10	iCBT	Information	PHQ-9	I and S
Hirsch et al [76], 2018	United Kingdom	Community	PHQ-9 ≥10	CBM^af^	Sham	PHQ-9	I
Hobfoll et al [77], 2016	United States	Veteran service	CED-D=8-25	iCBT	WL	CES-D-10^ag^	I and S
Høifødt et al [78], 2013	Norway	Clinic	BDI-II=14-29	iCBT	CAU	BDI-II	I and M
Holländare et al [79], 2011	Sweden	Community	MDD in remission; MADRS-S=7-19	iCBT	Monitoring	MADRS-S and BDI-II	S and M
Holst et al [80], 2018	Sweden	Clinic	Mild and moderate depression (DSM-IV); MADRS-S <35	iCBT	CAU	BDI-II	S and M
Hoorelbeke and Koster [81], 2017	Belgium	Community	MDD in remission	CCT	Active	BDI-II	I and S
Jelinek et al [82], 2020	Germany	Community	PHQ-9 >4	BA^ah^	Active	PHQ-9	I
Jelinek et al [82], 2020	Germany	Community	PHQ-9 >4	BA	CAU	PHQ-9	I
Johansson et al [83], 2019	Sweden	Clinic	MDD	iCBT	CAU	MADRS-S and HADS-D^ai^	S
Johansson et al [84], 2013	Sweden	Community	MDD	Psychodynamic therapy	Active	PHQ-9	S
Johansson et al [85], 2012	Sweden	Community	MADRS-S=15-35	Psychodynamic therapy	Active	BDI-II	S
Johansson et al [86], 2012	Sweden	Community	MADRS-S=15-35	iCBT	Active	BDI-II and MADRS-S	S
Kessler et al [87], 2009	United Kingdom	Clinic	BDI ≥14; MDD and MDE	iCBT	CAU	BDI-II	I and M
Kivi et al [88], 2014	Sweden	Clinic	MADRS-S <35; MDD	iCBT	CAU	BDI-II and MADRS-S	S
Kladnitski et al [89], 2020	Australia	Community	PHQ-9 >9; MDD	iCBT	CAU	PHQ-9	I and S
Klein et al [90], 2017	Germany	Multiple	PHQ-9=5-14	iCBT	CAU	PHQ-9 and HDRS-24^aj^	S, M, and L
Kok et al [91], 2015	The Netherlands	Multiple	MDD in remission	iCT	CAU	IDS-SR^ak^	I and S
Levesque et al [92], 2011	United States	Clinic	PHQ-9 ≥5; BDI=14-28	TTM^al^	CAU	BDI-II	M
Levin et al [93], 2011	United States	Clinic	Depressed mood; anhedonia	WW-CWD^am^	CAU	SCID and CES-D	I and M
Lindegaard et al [94], 2021	Sweden	Community	Elevated symptoms of depression	iCBT	WL	PHQ-9	I
Lokman et al [95], 2017	The Netherlands	Community	IDS-SR=14-38	CDMIs^an^	WL	IDS-SR	S
Loughnan et al [96], 2019	Australia	Community	PHQ-9 >9; EPDS ≥13	iCBT	CAU	EPDS and PHQ-9	I and S
Loughnan et al [97], 2019	Australia	Community	PHQ-9 ≥10	iCBT	CAU	EPDS and PHQ-9	I and S
Lu et al [98], 2021	Singapore	Clinic	Mild to moderate depressive symptoms	iCBT	CAU	PHQ-9	I
Lüdtke et al [99], 2018	Germany	Clinic	PHQ-9 >4	iCBT	CAU	PHQ-9	I
Lukas, and Berking [100], 2021	Germany	Community	PHQ-9 ≥5	Approach-avoidance biases and approach-avoidance modification training+CBT	WL	PHQ-9 and ADS^ao^	I and S
McCloud et al [101], 2020	United Kingdom	University	HADS^ap^ >8	Stress intervention	CAU	HADS	I and S
Meglic et al [102], 2010	Slovenia	Clinic	MDD (ICD-10^aq^); BDI-II >14	Information+therapist support	CAU	BDI-II	S
Meyer et al [103], 2015	Germany	Multiple	PHQ-9 >14	CAU+iCBT	CAU	PHQ-9	S
Milgrom et al [104], 2016	Australia	Community	EPDS=11-23	iCBT	Information	BDI-II	S
Mira et al [105], 2017	Spain	Community	BDI-II ≤28	iCBT	WL	BDI-II and ODSIS^ar^	S
Moberg et al [106], 2019	United States	Community	PHQ-8=5-14	iCBT	WL	DASS-21-D and PHQ-8	I
Monteiro et al [107], 2020	Portugal	Community	PDPI-R <5.5	iCBT	CAU	EPDS	I
Montero-Marin et al [108], 2016	Spain	Clinic	BDI-II=14-28	iCBT	CAU	BDI-II	S and L
Morgan et al [109], 2012	Australia	Community	Subthreshold depression symptoms	Cognitive training	Information	PHQ-9	I
Morgan et al [110], 2013	Australia	Community	Depressive symptoms	Cognitive training	Information	PHQ-9	I
Moritz et al [111], 2012	Germany	Community	Elevated depression symptoms	iCBT	WL	BDI-II	I
Mullin et al [112], 2015	Australia	University	Depressive symptoms (MINI)	iCBT	WL	PHQ-9	I
Newby et al [113], 2013	Australia	Community	Mild or moderate MDD; mixed anxiety and depressive disorder; PHQ-9 scores above clinical threshold	iCBT	WL	BDI-II and PHQ-9	S
Newby et al [114], 2014	Australia	Community	BDI-II >12	CBM or CB^as^ education	Sham	BDI-II	I
Noguchi et al [115], 2017	Japan	Community	CES-D ≥16; PHQ-9 ≥5	iCBT or sEFM^at^	WL	PHQ-9 and CES-D	I and S
Nygren et al [116], 2019	Sweden	Community	Depressive symptoms	iCBT	WL	BDI-II and PHQ-9	I
O’Mahen et al [117], 2014	United Kingdom	Community	EPDS >12	BA	CAU	EPDS	S
Oehler et al [118], 2020	Germany	Community	Mild to moderate depressive symptoms or dysthymia (MINI); PHQ-9=5-14	iCBT	Active	PHQ-9 and IDS-SR	I, S, and M
Ofoegbu et al [119], 2020	Nigeria	University	MDD	iCBT	CAU	BDI-II	S
Pfeiffer et al [120], 2020	United States	Clinic	PHQ-9 ≥10	iCBT	Sham	QIDS-SR	S
Phillips et al [121], 2014	United Kingdom	Workplace	PHQ-9 ≥2 on 5 items	iCBT	Sham	PHQ-9	I and S
Pictet et al [122], 2016	Switzerland	University	BDI ≥14	Imagery CBM	Sham	BDI-II	I
Pictet et al [122], 2016	Switzerland	University	BDI ≥14	Imagery CBM	WL	BDI-II	I
Pots et al [123], 2016	The Netherlands	Community	CES-D ≥10	ACT^au^	Active	CES-D	S
Pots et al [123], 2016	The Netherlands	Community	CES-D ≥10	ACT	WL	CES-D	S
Proudfoot et al [124], 2003	United Kingdom	Clinic	MDD; mixed anxiety and depression	iCBT	CAU	BDI-II	S and M
Proudfoot et al [125], 2004	Australia	Clinic	GHQ-12^av^ ≥4; CIS-R^aw^ ≥12	iCBT	CAU	BDI-II	I and S
Proudfoot et al [126], 2013	Australia	Community	DASS-21^ax^ ≥27-63	iCBT (blended with IPT^ay^, PST, and PP^az^)	WL	DASS-21	I
Proudfoot et al [126], 2013	Australia	Community	DASS-21 ≥27-63	iCBT (blended with IPT, PST, and PP)	Information	DASS-21	I and S
Reins et al [127], 2019	Germany	Community	MDD (SCID)	iCBT	Information	HRSD	I and S
Richards et al [128], 2015	Ireland	Community	BDI-II=14-28	iCBT	WL	BDI-II	I
Richards et al [129], 2020	United Kingdom	Clinic	Depressive symptoms	iCBT	CAU	PHQ-9	I
Ritvo et al [130], 2021	Canada	Clinic	BDI-II ≥14; MDD (MINI)	iCBT (mindfulness-based)+CAU	CAU	QIDS, HDRS, and BDI-II	S
Robichaud et al [131], 2020	Canada	Community	PHQ-9 ≥10	iCBT	CAU	PHQ-9	I
Roepke et al [132], 2015	United States	Community	CES-D ≥16	iCBT+PP	CAU	CES-D	I and S
Rollman et al [133], 2018	United States	Clinic	MDD	iCBT	CAU	PROMIS^ba^	S and M
Romero-Sanchiz et al [134], 2017	Spain	Clinic	BDI-II=14-28	iCBT	CAU	BDI-II	L
Rosso et al [135], 2016	United States	Community	MDD; PHQ-9=10-23	iCBT	Monitoring	HDRS and PHQ-9	S
Ruehlman and Karoly [136], 2021	United States	University	PHQ-8 ≥10	Transdiagnostic behavioral health skill training	WL	PHQ-8	I
Salamanca-Sanabria et al [137], 2020	Colombia	University	PHQ-9=10-19	iCBT	WL	PHQ-9	I
Salisbury et al [138], 2016	United Kingdom	Clinic	PHQ-9 ≥10; MDD (CIS-R)	iCBT	CAU	PHQ-9	S and M
Sandoval et al [139], 2017	United States	Community	MDD; PHQ-9 ≥10	PST	WL	BDI-II	I and S
Schure et al [140], 2019	United States	Community	PHQ-9 >5	iCBT	Information	PHQ-9	I
Segal et al [141], 2020	Canada	Clinic	MDD; PHQ-9=5-9	MBCT^bb^+CAU	CAU	PHQ-9	S and L
Smith et al [142], 2017	Australia	Clinic	PHQ-9=5-23	iCBT, CBT self-help book, and meditation self-help book	CAU	PHQ-9	S
Sun et al [143], 2021	China	Clinic	EPDS >9; PHQ-9 >4	Mindfulness-based intervention	Monitoring	EPDS	I and S
Terides et al [144], 2018	Australia	Community	PHQ-9 ≥5	iCBT	WL	PHQ-9	I
Titov et al [145], 2010	Australia	Community	MDD (MINI)	iCBT	WL	BDI-II and PHQ-9	I
Titov et al [146], 2013	Australia	Community	Self-identified depression	iCBT	WL	PHQ-9	I
Tönnies et al [147], 2021	Germany	Clinic	PHQ-9 >9	Video consultations	CAU	PHQ-9	S
Tønning et al [148], 2021	Denmark	Clinic	Unipolar depressive disorder (ICD-10)	MiCBT^bc^	CAU	HDRS-6^bd^/17, HAM-D6^be^, and BDI-II	S
Tulbure et al [149], 2018	United States	Community	BDI-II=14-50; MDD and dysthymia (DSM-IV)	iCBT; iCBT (religion-focused)	Monitoring	BDI-II	S
Twomey et al [150], 2014	Ireland	Clinic	Symptoms of depression	iCBT	CAU	DASS-21-D	I
Warmerdam et al [151], 2009	The Netherlands	Community	CES-D ≥16	iCBT	WL	CES-D	I and S
Yeung et al [152], 2018	China	Clinic	Significant depressive symptoms	iCBT	CAU	CES-D	I
Zwerenz et al [153], 2017	Germany	Clinic	BDI-II ≥13; MDD	iCBT+PP	Information	BDI-II	S

^a^PHQ-9: 9-item Patient Health Questionnaire.

^b^iCBT: internet-based or computerized cognitive behavioral therapy.

^c^IACT: internet-based acceptance and commitment therapy.

^d^I: immediate—<8 weeks since baseline.

^e^MDD: major depressive disorder.

^f^CIDI: Composite International Diagnostic Interview.

^g^MADRS-S: Montgomery-Åsberg Depression Rating Scale–Self-reported.

^h^BDI: Beck Depression Inventory.

^i^S: short term—9 to 24 weeks.

^j^M: medium term—25 to 48 weeks.

^k^QIDS: Quick Inventory of Depressive Symptomatology.

^l^CAU: care as usual.

^m^QIDS-SR: Quick Inventory of Depressive Symptomatology (Self-Report).

^n^HDRS: Hamilton Depression Rating Scale.

^o^BDI-II: Beck Depression Inventory-II.

^p^CES-D: Center for Epidemiologic Studies Depression Scale.

^q^WL: waitlist.

^r^SCID: Structured Clinical Interview for the Diagnostic and Statistical Manual of Mental Disorders, Fourth Edition.

^s^CCT: cognitive control training.

^t^DSM-IV: Diagnostic and Statistical Manual of Mental Disorders, Fourth Edition.

^u^iCT: internet-based cognitive therapy.

^v^PHQ-8: 8-item Patient Health Questionnaire.

^w^L: long term—>48 weeks.

^x^PSRT: Positive Self-Reference Training.

^y^DASS-21-D: Depression, Anxiety, and Stress Scale–21 items–Depression subscale.

^z^MINI: Mini International Neuropsychiatric Interview.

^aa^PDPI-R: Postpartum Depression Predictors Inventory–Revised.

^ab^EPDS: Edinburgh Postnatal Depression Scale.

^ac^iPST: internet-based problem-solving treatment.

^ad^MDE: major depressive episode.

^ae^PST: problem-solving training.

^af^CBM: cognitive bias modification.

^ag^CES-D-10: Center for Epidemiologic Studies Depression Scale Revised.

^ah^BA: web-based behavioral activation intervention.

^ai^HADS-D: Hospital Anxiety and Depression Scale (depression subscale).

^aj^HDRS-24: Hamilton Depression Rating Scale–24 items.

^ak^IDS-SR: Inventory of Depressive Symptomatology–Self-Report.

^al^TTM: transtheoretical model of behavior change.

^am^WW-CWD: wellness workshop CD-ROM based on the coping with depression intervention model.

^an^CDMI: complaint-directed mini-intervention.

^ao^ADS: The General Depression Scale.

^ap^HADS: Hospital Anxiety and Depression Scale.

^aq^ICD-10: International Classification of Diseases, 10^th^ Revision.

^ar^ODSIS: Overall Depression Severity and Impairment Scale.

^as^CB: cognitive behavioral education.

^at^sEFM: simplified emotional-focused mindfulness.

^au^ACT: acceptance and commitment therapy.

^av^GHQ-12: 12-item General Health Questionnaire.

^aw^CIS-R: Clinical Interview Schedule–Revised.

^ax^DASS-21: Depression, Anxiety, and Stress Scale–21 items.

^ay^IPT: interpersonal therapy.

^az^PP: positive psychology–based intervention.

^ba^PROMIS: Patient-Reported Outcomes Measurement Information System.

^bb^MBCT: mindfulness-based cognitive therapy.

^bc^MiCBT: mobile-based cognitive behavioral therapy.

^bd^HDRS-6: Hamilton Depression Rating Scale–6 items subscale.

^be^HAM-D6: Hamilton Depression Self-rating Scale–6 items.

### Study Quality

The overall quality of the included studies was not optimal according to the assessed results of the RoB 2 (Multimedia Appendix 2 [47-153]). Of the 107 publications, 11 (10.3%) were judged as having a high risk of bias. Most studies (91/107, 85%) were judged as having “some concerns,” as defined by the RoB 2. Most of these concerns were related to the lack of blinding, either to the participants or to the assessor, and therefore, this posed some concerns over the risk of bias associated with the measurement of the outcome. Only 4.7% (5/107) of the studies were considered high-quality.

### Characteristics of the Control Groups

A total of 113 control arms (some studies included multiple control arms) were coded in the included studies. In total, 6 distinct types were identified based on the contents and delivery modes stated in the articles.

#### Waitlist (26 Arms)

Participants in the waitlist control group waited for the commencement of the intervention. In cases where the study was conducted in a clinical setting, the control group was coded as a waitlist whenever the participants were restricted from contacting a therapist or receiving any interventions (psychological or pharmacological) provided by professionals (eg, general practitioners, psychologists, and psychiatrists) during the waiting period.

#### CAU (47 Arms)

Studies that used a CAU control group did not restrict the receipt of treatment; therefore, participants in these studies continued with their planned treatment. Note that the waitlist control groups in studies on health care settings (eg, mental health services, clinics, or primary care) were considered as CAU when there was no mention of restriction of care.

#### Information (16 Arms)

Participants in the information control group received information in addition to their usual care. This information could be psychoeducational material; health tips; or anything provided on websites, on a pamphlet, or in other media.

#### Active (10 Arms)

Participants received procedures that were expected to bring about improvements on their depression. These included attending a discussion forum, expressive writing exercises, and nondirective supportive therapy provided by an assigned therapist who was independent of their usual care.

#### Sham (10 Arms)

A sham control group was highly similar to the comparator intervention but was intentionally designed to remove active therapeutic elements. For example, in cognitive training, a sham control condition used the same procedure as the intervention group but with modified stimuli.

#### Monitoring (4 Arms)

Participants in a monitoring control group were constantly assessed on their depressive symptoms during the study period in addition to the major assessment time points. For instance, they completed questionnaires repeatedly or received prompted messages asking about their mood throughout the study.

### Assessment Time and Retention

The mean time interval was 8.88 (SD 6.06) weeks between baseline and the postassessment time point, 21.87 (SD 12.5) weeks between baseline and the first follow-up, and 41.38 (SD 30.31) weeks between baseline and the second follow-up. The mean retention rate was 79.54% (SD 17.05%) at the postassessment time point, 75.13% (SD 17.56%) at the first follow-up, and 66.76% (SD 16.90%) at the second follow-up. The retention rates did not differ significantly across control types at the postassessment time point (*F*_5,92_=0.8; *P*=.53) and at the first (*F*_5,48_=0.7; *P*=.63) and second (*F*_5,14_=0.8; *P*=.57) follow-ups.

### Time-Dependent Effects on Depressive Symptoms

#### Overview

**Figure 2:**
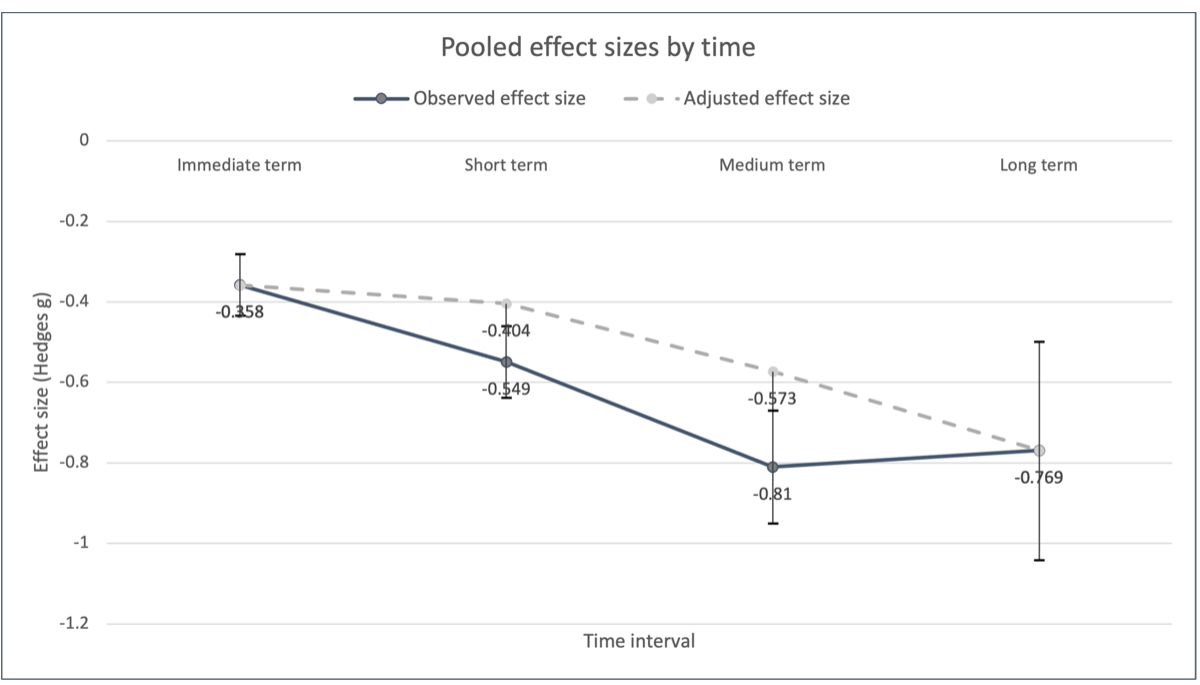
Effect sizes by time since baseline assessment.

The primary focus of this study was the pre-post changes in depressive symptoms across different time durations among the controlled participants. Table 2 summarizes the detailed results of the meta-analysis. Figure 2 shows the trend in effects across the 4 time intervals outlined in the following sections. See Multimedia Appendix 3 [47-153] for the forest plots.

**Table 2:** Effect summary of the meta-analysis.^a^

			Arms, n		Participants, N		Hedges *g* (95% CI)		Q		*I* ^2^		Q_between_			*P* value	
**Immediate (≤8 weeks)**																	
	Overall effect		72	7400		−0.358 (−0.434 to −0.281)		802.62		91.15		N/A^b^			N/A		
	**By control type**													10.48^c^			.03
		WL^d^	23	2038		−0.234 (−0.348 to −0.120)		159.12		86.17							
		CAU^e^	23	1703		−0.390 (−0.522 to −0.257)		182.56		87.95							
		Information	12	2612		−0.527 (−0.703 to −0.350)		199.25		94.48							
		Sham	9	650		−0.462 (−0.673 to −0.251)		44.90		82.18							
		Active	4	313		−.207 (−0.458 to −0.043)		14.43		79.21							
	**By severity**													19.65^f^			<.001
		Mild	11	854		−0.122 (−0.285 to 0.041)		64.31		84.45							
		Moderate	38	3793		−0.317 (−0.406 to −0.228)		289.84		87.23							
		Severe	21	2415		−0.572 (−0.699 to −0.444)		168.04		88.10							
**Short term (9-24 weeks)**																	
	Overall effect		67	6927		−0.549 (−0.638 to −0.460)		915.17		92.79		N/A			N/A		
	**By control type**													10.06			.07
		WL	12	1132		−0.291 (−0.478 to −0.104)		111.22		90.11							
		CAU	29	3402		−0.652 (−0.814 to −0.489)		608.54		95.40							
		Information	11	1138		−0.585 (−0.756 to −0.414)		82.83		87.93							
		Active	9	582		−0.543 (−0.777 to −0.310)		63.58		87.42							
		Monitoring	4	192		−0.519 (−0.702 to −0.335)		5.07		40.83							
		Sham	2	481		−0.614 (−0.746 to −0.483)		2.07		51.59							
	**By severity**													9.02^c^			.01
		Mild	10	1064		−0.241 (−0.486 to 0.004)		156.23		94.24							
		Moderate	34	3436		−0.602 (−0.712 to −0.493)		324.53		89.83							
		Severe	18	1695		−0.696 (−0.880 to −0.511)		232.66		92.69							
**Medium term (25-48 weeks)**																	
	Overall effect		24	2861		−0.810 (−0.950 to −0.670)		291.46		92.11		N/A			N/A		
	**By control type**													7.85^c^			.02
		CAU	14	1900		−0.950 (−1.161 to −0.739)		228.74		94.32							
		Information	3	381		−0.407 (−0.758 to −0.055)		24.82		91.94							
		Active	4	343		−0.653 (−0.871 to −0.435)		11.13		73.06							
	**By severity**													7.69^c^			.02
		Mild	2	115		−0.473 (−0.694 to −0.251)		1.55		35.44							
		Moderate	13	1324		−0.894 (−1.132 to −0.657)		181.71		93.40							
		Severe	8	1246		−0.808 (−0.997 to −0.619)		69.37		89.91							
**Long term (>48 weeks)**																	
	Overall effect		9	1641		−0.769 (−1.041 to −0.498)		207.39		96.14		N/A			N/A		
	**By control type**																
		CAU	7	1517		−0.827 (−1.133 to −0.521)		179.57		96.66		N/A			N/A		
	**By severity**													1.18			.28
		Moderate	5	553		−0.775 (−1.332 to −0.219)		131.04		96.95							
		Severe	2	354		−1.091 (−1.210 to −0.972)		.57		N/A							

^a^A subgroup of <2 studies was not included in the subgroup analysis.

^b^N/A: not applicable.

^c^*P<*.05.

^d^WL: waitlist.

^e^CAU: care as usual.

^f^*P*<.001.

#### Immediate Effect

The results showed that, within the first 8 weeks since the baseline assessment, the overall effect of the control groups, as estimated among 67.3% (72/107) of the studies with a total of 7400 participants, was *g=*−0.358 (95% CI −0.434 to −0.281), indicating a small to moderate effect. Funnel plot symmetry (Multimedia Appendix 4) and a nonsignificant ranked correlation (*P*=.95) and Egger regression (*P*=.14) suggested no significant publication bias for the effect size obtained.

#### Short-term Effect

The overall effect during weeks 9 to 24 weeks since baseline was estimated among 62.6% (67/107) of the studies with a total of 6927 participants, and the pooled effect size was *g=*−0.549 (95% CI −0.638 to −0.460), indicating a moderate effect. Minimal funnel plot asymmetry was observed (Multimedia Appendix 4), and the Egger regression was significant (*P*=.02), indicating publication bias. In the trim-and-fill procedure by Duval and Tweedie [46], 15 effect sizes were imputed to the right of the mean using the fixed-effects model, resulting in an adjusted effect size of *g=*−0.404 (95% CI −0.496 to −0.312).

#### Medium-term Effect

Within the 25th to 48th weeks, the effect across 22.4% (24/107) of the studies (n*=*2861 participants) was estimated to be *g=*−0.810 (95% CI −0.950 to −0.670), which is considered a large effect. A significant Egger regression (*P*=.008) and funnel plot asymmetry (Multimedia Appendix 4) suggested substantial publication bias. A total of 9 effect sizes were imputed to the right of the mean by looking for missing studies using the fixed-effects model, resulting in an adjusted effect size of *g=*−0.573 (95% CI −0.720 to −0.426).

#### Long-term Effect

Finally, the overall effect flattened approximately a year later beyond 48 weeks among 8.4% (9/107) of the studies with a pooled sample size of 1641, maintaining a close-to-large effect range of *g=*−0.769 (95% CI −1.041 to −0.498). Funnel plot symmetry (Multimedia Appendix 4) and a nonsignificant Egger regression (*P*=.60) suggested an absence of publication bias.

### Differential Effect of Control Type

**Figure 3:**
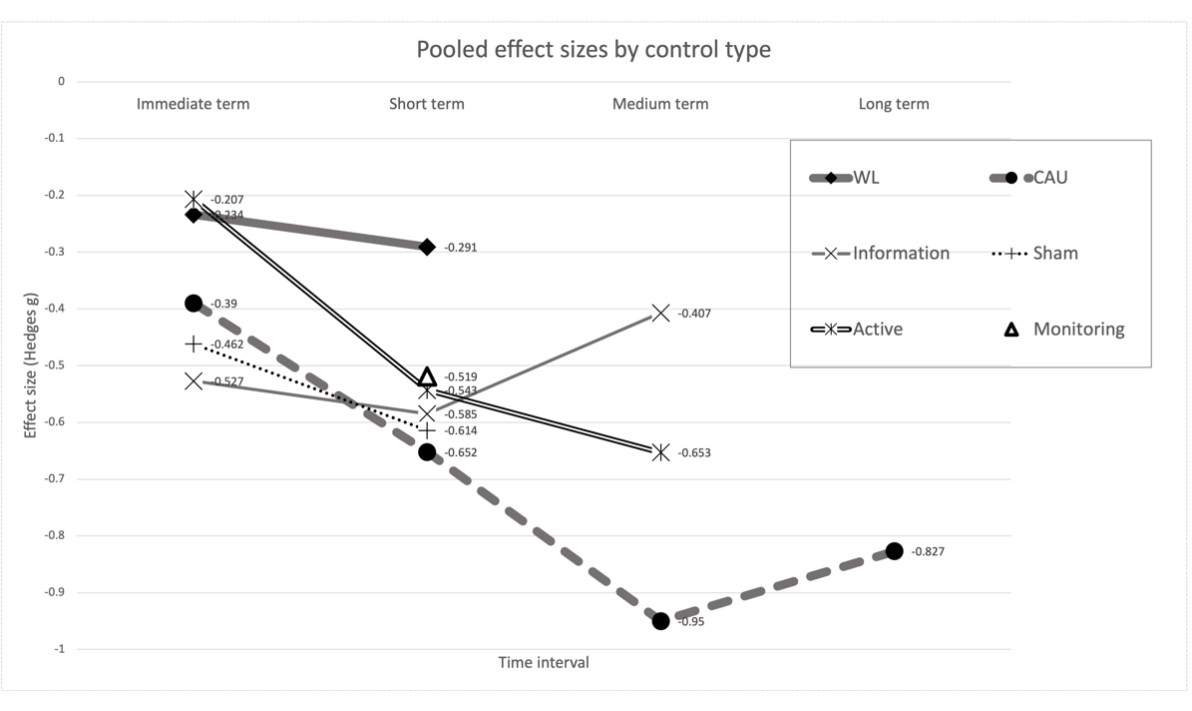
Effect sizes by control type. CAU: care as usual; WL: waitlist.

Considering the differential effect of control type, the difference between subgroups was significant in the immediate (*Q*_between_=10.48; *P*=.03) and medium term (*Q*_between_=7.85; *P*=.02) but not in the short term (*Q*_between_=10.06; *P*=.07), and there were insufficient data to compare in the long term. As illustrated in Figure 3, the CAU control type (thick dashed line and circle markers) produced the strongest effect overall after 8 weeks, reaching its peak of *g=*−0.950 (95% CI −1.161 to −0.739) in the medium term. The waitlist control (thick solid line and diamond markers) produced relatively smaller effects as compared with the other control types.

The effect of active controls (thin solid line and star markers) was small shortly after the baseline assessment (*g=*−0.207) but strengthened to a moderate effect in the medium term (*g=*−0.653). Sham controls (thin dotted line and “+” markers) consistently produced close-to-moderate effects from the immediate term (*g=*−0.462) to the short term (*g=*−0.614).

Although most control types have accumulating effects over time, information controls (thin solid line and cross markers) showed a drop from a moderate effect (*g=*−0.585) to a small effect (*g=*−0.407) in the medium term. Finally, only 3.7% (4/107) of the studies (n*=*192 participants) used a monitoring control type (triangle marker), which was assessed in the short term in all cases. The pooled effect of these monitoring controls was moderate (*g=*−0.519).

### Differential Effect of Severity

**Figure 4:**
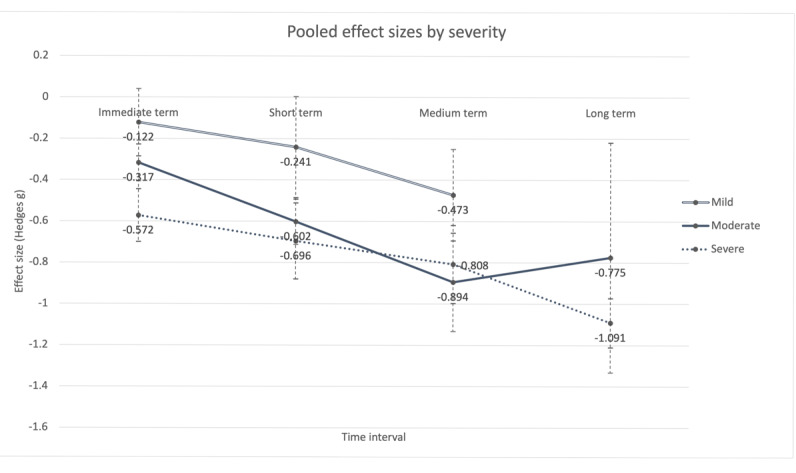
Effect sizes by severity.

Considering the differential effect of severity, subgroup analysis results showed significant differences across levels of severity in the immediate (*Q*_between_=19.65; *P*<.001), short (*Q*_between_=9.02; *P*=.01), and medium (*Q*_between_=7.69; *P*=.02) term but not in the long term (*Q*_between_=1.18; *P*=.28). As illustrated in Figure 4, studies in which there were lower levels of severity at baseline appeared to produce smaller effects in reducing depressive symptoms in the immediate, short, and long term but not in the medium term.

Depressive symptoms did not change significantly among studies in which there were mild levels of baseline symptoms in the immediate (*P*=.14) and short (*P*=.05) term. Studies in which there were moderate levels of depressive symptoms were estimated to produce the largest effect on symptom reduction in the medium term (*g=*−0.894). In addition, an opposite trend between studies in which there were moderate (solid line in Figure 4) and severe (dotted line) levels of symptoms in the medium and long term was observed. Specifically, the effect started to shrink in the moderate-severity subgroup but started to grow in the severe subgroup beyond 48 weeks.

### Meta-regression on Demographics

As the heterogeneity was very high (>75%) even after subgrouping, a series of meta-regression analyses were performed to examine whether participants’ age, gender, and education level accounted for some of the heterogeneity. As shown in Table 3, in terms of unique variance, female participants (*z*=−2.37; *P*=.02) and education level (*z*=1.98; *P*=.047) in the short term as well as age (*z*=2.15; *P*=.03) in the medium term appeared to significantly explain some variance in the effect sizes. Specifically, the coefficients indicated that the higher the proportion of female participants and the lower the proportion of participants who had a higher education in a study, the smaller the effect in the short term; studies with older participants were found to obtain larger effect sizes in the medium term. However, when considering all covariates collectively, none of the regression models were significant, indicating that the moderating effect of the covariates was very limited and that these covariates together were unable to explain the variance in effect sizes at each of the time intervals.

**Table 3:** Summary of meta-regression results.

Time interval and variable			*b* (SE)	95% CI		*z*		*P* value
**Immediate^a^**								
	Age	−0.002 (0.01)		−0.014 to 0.010	−0.32		.75	
	Female participants	−0.005 (0.003)		−0.010 to 0.001	−1.66		.01	
	Higher education	−0.001 (0.003)		−0.004 to 0.006	0.03		.79	
**Short term^b^**								
	Age	−0.005 (0.01)		−0.019 to 0.008	−0.76		.45	
	Female participants	−0.008 (0.003)		−0.014 to −0.001	−2.37^c^		.02	
	Higher education	−0.005 (0.003)		<−0.001 to 0.001	1.98^c^		.047	
**Medium term^d^**								
	Age	0.04 (0.02)		0.004 to 0.081	2.15^c^		.03	
	Female participants	−0.003 (0.01)		−0.016 to 0.011	−0.41		.68	
	Higher education	0.005 (0.01)		−0.005 to 0.014	0.94		.35	
**Long term^e^**								
	Age	0.138 (0.10)		−0.056 to 0.332	1.40		.16	
	Female participants	−0.001 (0.04)		−0.072 to 0.071	−0.01		.99	
	Higher education	−0.015 (0.02)		−0.051 to 0.021	−0.81		.42	

^a^Model summary: *τ*^2^=0.091; *R*^2^=17%; *Q*(3)=3.08; *P*=.38.

^b^Model summary: *τ*^2^=0.110; *R*^2^=7%; *Q*(3)=7.41; *P*=.06.

^c^*P<*.05.

^d^Model summary: *τ*^2^=0.102; *R*^2^<.001%; *Q*(3)=6.77; *P*=.08.

^e^Model summary: *τ*^2^=0.156; *R*^2^<.001%; *Q*(3)=2.23; *P*=.53.

## Discussion

### Principal Findings

This meta-analytic study examined the magnitude of the effects associated with different types of control conditions in DPI trials targeting depressive symptoms among adults. On the basis of the 107 included studies, we observed a U-shaped trend in depressive symptom reduction over time. Specifically, the effect sizes were small to moderate (*g=*−0.358) in the first 8 weeks since the baseline assessment; then, a moderate effect (*g=*−0.549) was noted between the 9th and 24th weeks, which was approximately half a year later. After adjusting for publication bias, this short-term effect was reduced to a small to moderate effect (*g=*−0.404). Within the second half of the same year (25th to 48th weeks), the effect peaked at *g=*−0.810 (adjusted: *g=*−0.573), indicating a large (moderate after adjustment) effect. Although the effect shrank slightly beyond 48 weeks (a year later), it was maintained at a close-to-large effect range (*g=*−0.769).

To the best of our knowledge, this study is the first attempt that looked at the longitudinal trajectory of depressive symptoms among control participants in DPI studies. Undoubtedly, the effect sizes obtained in the control conditions were generally smaller than those of the intervention conditions that were reported [22-27]. However, the findings concur with those of previous studies on face-to-face interventions that, even without active interventions, symptomatic remission is possible [31,33,34]. Considering people in the waitlist control group who were highly restricted on the receiving of treatment procedures, the reduction in depressive symptoms reflected a collective variance explained by spontaneous recovery and regression to the mean, at least to some extent. Although this subtype of control yielded relatively weaker effects than other control conditions given its passivity, the improvement was still significant and meaningful.

Regarding the suspicion of Furukawa et al [35] that people being assigned to a waitlist control may hinder their self-healing behaviors and, thus, experience a nocebo effect (ie, worsening of depression while waiting), our findings did not support this claim as waitlist control groups in our meta-analysis reduced depressive symptoms significantly within the first 24 weeks. However, given that the authors separated waitlist from no-treatment control groups, whereas we did not identify a purely no-treatment condition, our findings could not refute the nocebo hypothesis entirely. The authors of that study investigated face-to-face CBT; it may also be possible that the nocebo effect only applies to people who are willing to receive face-to-face psychotherapy but not to those receiving DPIs.

Moreover, the results concerning CAU were difficult to interpret given the broad definition of “usual treatment” used. Of the 44 studies that used a CAU control group, surprisingly, only 2 (5%) [71,80] detailed the care procedures involved. After all, CAU has been at best summarized as a blend of psychosocial and pharmacotherapy treatments. Regardless of the heterogeneity, it was suggested that the differential effect among subtypes of CAU was not significant [33]; therefore, we interpreted the effects of CAU as a collective therapeutic strength of existing services, and encouragingly, CAU was able to produce strong improvement in symptoms of depression in the medium term (*g=*−0.956), which is comparable with the low end of the within-group effect sizes as estimated in the active intervention arms in DPI studies [26].

The information control was an interesting type of control to consider given that reading educational materials is sometimes regarded as a stand-alone passive intervention. Our results showed that the information control was quite a strong intervention that produced a moderate effect immediately after the intervention period and in the short term. This is in line with previous meta-analytic findings showing that psychoeducational interventions were able to reduce symptoms of depression [154]. The effect of information controls shrank after 6 months in our study, indicating that the effect of passively receiving mental health–related information may dissipate over time. Although psychoeducation generally forms the core of most large-scale campaigns, for example, public awareness campaigns that disseminate mental health information, campaign holders may consider hosting booster sessions or setting up reminder prompts from time to time to maintain the effect in the long run.

Other control types, including sham, monitoring, and active controls, were also found to have moderate effects in the short term, and the effect was larger than that of waitlist controls. We acknowledge that the number of studies in each control category was very small and may have been subject to a small study bias. Nonetheless, the pattern of results points to the possibility that, regardless of the content of the control condition that the participants received, engagement or taking some action may help alleviate depression under the principle of behavioral activation, and these approaches fared better than simply waiting.

Subgroup analysis results also showed that, regardless of baseline severity, depressive symptoms reduced over time, although participants who started off with mild levels of severity consistently recorded a smaller effect size, possibly because of the floor effect. Studies in which there were moderate levels of depressive symptoms at baseline were associated with a steeper drop in symptoms until the medium term, and symptoms then bounced back in the long term. This implies that the participants in those studies may have reached the maximum potential for spontaneous recovery at approximately 48 weeks. Finally, the strongest effect noted in the severe subgroup beyond 48 weeks could be attributed to the exceptional efficacy of the CAU control type that dominated in this set of studies being analyzed.

### Limitations

As with any study, this meta-analysis had several limitations. First, the use of pre-post (within-subject) SMD may result in biased outcomes [155]. A reason is that the estimation of pre-post SMD depends on the correlation between pre-post outcomes, which is often not reported in studies. In our meta-analysis, we used a conservative correlation of *r*=0.59, which was based on the median within-group correlation found across 123 studies in the study by Balk et al [42]. However, given that each study may vary in the correlation estimate, there may be measurement error. Second, the quality of the included studies was not optimal. Almost all the studies (91/107, 85%) demonstrated some concerns associated with blinding issues given the nature of psychological interventions, be they face to face or digital [156], and possible underreporting of blinding procedures [157]. Only 4.7% (5/107) could be judged as high-quality, which was disappointing but understandable. It has been suggested that studies of poor quality generally inflate their effect sizes [158,159]; therefore, our interpretation of the findings should be taken with caution. Although it is difficult to truly blind participants in DPIs, blinding other key persons, including data managers, statisticians, and conclusion makers, is comparatively practical. Nonetheless, researchers should make an effort to blind all parties whenever possible. Mataix-Cols and Andersson [160] recently detailed 10 practical recommendations on blinding that future DPI studies should attend to.

In addition, the high degree of heterogeneity (>75%) among almost all sets of analyses made interpretation very difficult [44]. Despite our efforts to regress the overall effects on age, gender, and education, these individual characteristics were insufficient to explain the heterogeneity. A possible reason for this high heterogeneity may be the use of a relatively broad inclusion criterion that encompassed people with any depressive symptoms along the spectrum, even though studies targeting obvious comorbid conditions, for instance, physical medical conditions, posttraumatic stress disorders, and psychotic disorders, had already been excluded. Nonetheless, we included studies with comorbid anxiety given that it is highly common and can enhance the external validity of our findings. Future analyses may try to narrow down the depression subtypes and focus on people without comorbid conditions.

Some may believe that there is no point in pooling effect sizes of such different conditions and that effect sizes should not be pooled in case of substantial statistical heterogeneity. However, we argue that those who agree to participate in a DPI RCT represent an overarching group of individuals that share a similar mentality, for instance, being open to receiving interventions and self-motivated to change. Moreover, the high heterogeneity provided important insights on the documentation of control procedures and contents. We also recommend future trials to gather information regarding external help-seeking behaviors exhibited by control participants during the study period.

### Implications

Despite these limitations, this study is the first comprehensive meta-analytic review that focuses primarily on the control groups of DPI studies. It provides valuable information regarding the magnitude of improvements in depressive symptoms in various control conditions over time. We quantified the within–control-group effect sizes among a fairly large number of DPI RCTs. Our findings have important implications for intervention research and practice. In psychosocial intervention research, studies that adopt a quasi-experimental design might overstate the treatment efficacy by not being able to account for the highly significant nontreatment effects, as quantified in this study. To uphold scientific rigor, we advocate that an RCT design should be perceived as the minimum requirement, not a high standard, for conducting intervention research. Strictly speaking, a quasi-experimental design should be abandoned for this type of research.

This study also provided evidence that depressive symptoms generally decrease over time. The severity of the symptoms reduced significantly within a few weeks even without structural treatment. Here, we must clarify that it is not our intention to discourage help-seeking behaviors or discredit the values of established evidence-based treatments. Instead, we hope that, by informing of a natural remitting trajectory of depression, researchers and practitioners and, most importantly, people who are experiencing various levels of depressive symptoms themselves do not see depression as a never-changing condition and negate the possibility of spontaneous recovery. They may consider adopting watchful waiting when active mental health services are not readily available given that waitlists were found to be efficacious in the short term.

### Conclusions

In summary, this meta-analysis focused on the effects of control groups in DPI studies. The results concurred with previous findings on face-to-face psychotherapy studies that control conditions were able to produce small to moderate reductions in depressive symptoms within and beyond 48 weeks. Future studies should continue to investigate the nonspecific effects in intervention studies and explore meaningful moderators to explain the heterogeneity in DPI studies.

## Appendix

Multimedia Appendix 1 Search strategy and severity cutoffs.

Multimedia Appendix 2 Quality assessment (Cochrane risk-of-bias tool for randomized trials version 2).

Multimedia Appendix 3 Forest plots.

Multimedia Appendix 4 Funnel plots.

## Abbreviations

CAU: care as usual
CBT: cognitive behavioral therapy
DPI: digital-based psychological intervention
PRISMA: Preferred Reporting Items for Systematic Reviews and Meta-Analyses
PRISMA-P: Preferred Reporting Items for Systematic Review and Meta-Analysis Protocols
RCT: randomized controlled trial
RoB 2: Cochrane risk-of-bias tool for randomized trials version 2
SMD: standardized mean difference
