# Supplementary material for: Time-Dependent Changes in Depressive Symptoms Among Control Participants in Digital-Based Psychological Intervention Studies: Meta-analysis of Randomized Controlled Trials
Source: J Med Internet Res. 2023 Apr 12;25:e39029. doi: 10.2196/39029 (PMC10134030; doi:10.2196/39029)
Supplement: Multimedia Appendix 1 [file jmir_v25i1e39029_app1.pdf]

Multimedia Appendix 1. Search strategy and severity cutoffs.

1.1 Search strategy

Database	Terms	Additional filers
Pubmed	(("internet*"[TW] OR "web"[TW] OR "online*"[TW] OR "computer*"[TW]) OR ("mobile"[TW] OR "smartphone *"[TW] OR "phone*"[TW] OR "app"[TW] OR "mobile-app*"[TW])) AND ("treatment*"[TW] OR "therap*"[TW] OR "intervention*"[TW]) AND ("depress*"[TW] OR "mood*"[TW] OR "mdd"[TW] OR "mde"[TW]) AND ("RCT"[TW] OR "random*"[TW] OR "clinical trial"[TW]) NOT ("protocol*"[TW] OR "systematic review*"[TW] OR "review*"[TW]OR "meta*"[TW] OR "child*"[TW] OR "adolesc*"[TW] OR "elder*"[TW] OR "senior*"[TW] OR "older*"[TW])	English Journal articles
ProQuest	AB,TI,IF("internet*" OR "web" OR "online*" OR "computer*" OR "mobile*" OR "smartphone*" OR "phone*" OR "app" OR "mobile-app*") AND AB,TI,IF("treatment*" OR "therap*" OR "intervention*") AND AB,TI,IF("depress*" OR "mood*" OR "mdd" OR "mde") AND AB,TI,IF("RCT" OR "random*" OR "clinical trial") NOT AB,TI,IF("protocol*" or ("systematic reviews") or "review*" or "meta*" or "child*" or "adolesc*" or "elder*" or "senior*" or "older*")	Peer-reviewed Article Scholarly journal English
Web of Science	(TS=("internet*" OR "web" OR "online*" OR "computer*" OR "mobile*" OR "smartphone*" OR "phone*" OR "app" OR "mobile-app*") AND TS=("treatment*" OR "therap*" OR "intervention*") AND TS=("depress*" OR "mood*" OR "mdd" OR "mde") AND TS=("RCT" OR "random*" OR "clinical trial")) NOT TS=("protocol*" or "systematic review*" or "review*" or "meta*" or "child*" or "adolesc*" or "elder*" or "senior*" or "older*")	English Article
OVID (EMBASE MEDLINE PSYCINFO)	("internet*" or "web" or "online*" or "computer*" or "mobile*" or "smartphone*" or "phone*" or "app" or "mobile-app*").ab,kw,ti. AND ("treatment*" or "therap*" or "intervention*").ab,kw,ti. AND ("depress*" or "mood*" or "mdd" or "mde").ab,kw,ti. AND ("RCT" or "random*" or "clinical trial").ab,kw,ti. NOT ("protocol*" or "systematic reviews" or "review*" or "meta*" or "child*" or "adolesc*" or "elder*" or "senior*" or "older*").ab,kw,ti.	English Peer-reviewed Journals

1.2 Severity of depressive symptoms cutoff scores and references

Tool	Minimal - Mild	Moderate	Moderately sever - Severe	Reference
PHQ-9	0-9	10-14	15+	Kroenke, K., Spitzer, R. L., & Williams, J. B. (2001). The PHQ-9: validity of a brief depression severity measure. Journal of general internal medicine, 16(9), 606-613.
PHQ-8	0-9	10-14	15+	Mattsson, M., Sandqvist, G., Hesselstrand, R., Nordin, A., & Boström, C. (2020). Validity and reliability of the Patient Health Questionnaire-8 in Swedish for individuals with systemic sclerosis. Rheumatology International, 40(10), 1675-1687.
BDI-II	0-18	19-29	30+	Beck, Aaron T, Steer, Robert A, & Carbin, Margery G. (1988). Psychometric properties of the Beck Depression Inventory: Twenty-five years of evaluation. Clinical Psychology Review, 8(1), 77-100.
PROMIS (T-score)	55	60	65	Kroenke, K., Stump, T. E., Chen, C. X., Kean, J., Bair, M. J., Damush, T. M., Krebs, E. E., & Monahan, P. O. (2020). Minimally important differences and SEVERITY thresholds are estimated for The PROMIS depression scales from three randomized clinical trials. Journal of Affective Disorders, 266, 100–108. https://doi.org/10.1016/j.jad.2020.01.101
DASS-21-D	0-13	14-20	21+	Lovibond, S.H. & Lovibond, P.F. (1995). Manual for the Depression Anxiety & Stress Scales. (2nd Ed.)Sydney: Psychology Foundation.
EPDS	0-13	14-18	19+	McCabe-Beane, J. E., Segre, L. S., Perkhounkova, Y., Stuart, S., & O'Hara, M. W. (2016). The identification of severity ranges for the Edinburgh Postnatal Depression Scale. Journal of Reproductive and Infant Psychology, 34(3), 293-303.
HRSD/HAMD-17	0-16	17-23	24+	Zimmerman, M., Martinez, J. H., Young, D., Chelminski, I., & Dalrymple, K. (2013). Severity classification on the Hamilton Depression Rating Scale. Journal of affective disorders, 150(2), 384–388. https://doi.org/10.1016/j.jad.2013.04.028
HRSD/HAMD-24	0-19	20-34	35+	Pan, S., Liu, Z. W., Shi, S., Ma, X., Song, W. Q., Guan, G. C., ... & Lv, Y. (2017). Hamilton rating scale for depression-24 (HAM-D24) as a novel predictor for diabetic microvascular complications in type 2 diabetes mellitus patients. Psychiatry research, 258, 177-183.
HADS	8-10	11-14	15+	Stern, A. F. (2014). The hospital anxiety and depression scale. Occupational medicine, 64(5), 393-394.

QIDS-SR	0-10	11-15	16+	Trivedi, M. H, Rush, A. J, Ibrahim, H. M, Carmody, T. J, Biggs, M. M, Suppes, T, . . . Kashner, T. M. (2004). The Inventory of Depressive Symptomatology, Clinician Rating (IDS-C) and Self-Report (IDS-SR), and the Quick Inventory of Depressive Symptomatology, Clinician Rating (QIDS-C) and Self-Report (QIDS-SR) in public sector patients with mood disorders: A psychometric evaluation. Psychological Medicine, 34(1), 73-82.
IDS-SR	0-25	26-38	39+	Rush, A. (2003). The 16-Item Quick Inventory of Depressive Symptomatology (QIDS), clinician rating (QIDS-C), and self-report (QIDS-SR): A psychometric evaluation in patients with chronic major depression. Biological Psychiatry., 54(5), 573
MADRS-S	0-19	20-34	35+	Müller, Matthias J, Himmerich, Hubertus, Kienzle, Barbara, & Szegedi, Armin. (2003). Differentiating moderate and severe depression using the Montgomery–Åsberg depression rating scale (MADRS). Journal of Affective Disorders, 77(3), 255-260.
CES-D	0-20	21-25	25+	Unützer, J., Patrick, D. L., Marmon, T., Simon, G. E., & Katon, W. J. (2002). Depressive symptoms and mortality in a prospective study of 2,558 older adults. The American journal of geriatric psychiatry, 10(5), 521-530.

MADRS-S: The Montgomery and Åsberg Depression Rating Scale – Self-reported; QIDS-SR: Quick Inventory of Depressive Symptomatology (Self-report); PHQ-9/8: Patient Health Questionnaire – 9 items/8 items; BDI-II: Beck Depression Inventory-II; CES-D: Center for Epidemiologic Studies Depression Scale; DASS-21-D: Depression, Anxiety and Stress Scale – 21 items – Depression subscale; EPDS: Edinburgh Postnatal Depression Scale; IDS-SR: Inventory of Depressive Symptomatology – Self-report; HADS: Hospital Anxiety and Depression Scale; HDRS/HAMD: Hamilton Depression Rating Scale; PROMIS: Patient-Reported Outcomes Measurement Information System
