# Supplementary material for: Time-Dependent Changes in Depressive Symptoms Among Control Participants in Digital-Based Psychological Intervention Studies: Meta-analysis of Randomized Controlled Trials
Source: J Med Internet Res. 2023 Apr 12;25:e39029. doi: 10.2196/39029 (PMC10134030; doi:10.2196/39029)
Supplement: Multimedia Appendix 2 [file jmir_v25i1e39029_app2.pdf]

Multimedia Appendix 2 – Quality Assessment (ROB-2)

Study, year	Randomization process	Deviations from intended interventions	Missing outcome data	Measurement of the outcome	Selection of the reported result	Overall Bias
Al-Alawi et al [47], 2021	Low	High	Low	Some concerns	Some concerns	High
Andersson et al [48], 2005	Low	Some concerns	Low	Some concerns	Some concerns	Some concerns
Beevers et al [49], 2017	Low	Low	Low	Some concerns	Low	Some concerns
Berger et al [50], 2018	Low	Low	Low	Some concerns	Low	Some concerns
Birney et al [51], 2016	Low	Low	Low	Some concerns	Low	Some concerns
Bohlmeijer et al [52], 2021	Some concerns	Low	Low	Some concerns	Low	Some concerns
Boschloo et al [53], 2019	Some concerns	Some concerns	Low	Low	Low	Some concerns
Braun et al [54], 2021	Low	Low	Low	Some concerns	Low	Some concerns
Browning et al [55], 2012	Some concerns	Low	Low	Low	Low	Some concerns
Buntrock et al [56], 2015	Some concerns	Low	Low	Some concerns	Low	Some concerns
Calkins et al [57], 2015	Some concerns	Low	Low	Some concerns	Low	Some concerns
Choi et al [58], 2012	Some concerns	Low	Low	Some concerns	Low	Some concerns
Clarke et al [59], 2005	Some concerns	Low	Low	Some concerns	Some concerns	Some concerns
Clarke et al [60], 2009	Some concerns	Low	Low	Some concerns	Low	Some concerns
Dainer-Best et al [61], 2018	Low	Low	Low	Low	Low	Low
Day et al [62], 2013	Low	Low	Low	Some concerns	Low	Some concerns
De Graaf et al [63], 2011	Low	Some concerns	Low	Some concerns	Low	Some concerns
Eriksson et al [64], 2017	Low	High	Low	Some concerns	Some concerns	High
Flygare et al [65], 2020	Low	Low	Low	Low	Some concerns	Some concerns
Fonseca et al [66], 2020	Some concerns	Low	Low	Some concerns	Low	Some concerns
Geraedts et al [67], 2014	Low	Low	Low	Some concerns	Low	Some concerns
Gilbody et al [68], 2015	Low	Low	Low	Some concerns	Low	Some concerns
Gili et al [69], 2020	Low	Some concerns	Low	Some concerns	Some concerns	Some concerns
Hallford et al [70], 2021	Low	Low	Low	Some concerns	Low	Some concerns
Hallgren et al [71], 2016	Low	Low	Low	Some concerns	Low	Some concerns
Hange et al [72], 2017	Low	Low	Low	Some concerns	Low	Some concerns
Harrer et al [73], 2021	Low	Low	Low	Some concerns	Low	Some concerns
Hatcher et al [74], 2018	Low	High	Low	Some concerns	Low	High
Heim et al [75], 2021	Some concerns	Low	Some concerns	Some concerns	Low	Some concerns
Hirsch et al [76], 2018	Some concerns	High	Low	Some concerns	High	High
Hobfoll et al [77], 2016	Some concerns	Low	Low	Some concerns	Low	Some concerns

Høifødt et al [78], 2013	Low	Low	Low	Some concerns	Low	Some concerns
Holländare et al [79], 2011	Some concerns	Low	Low	Some concerns	Low	Some concerns
Holst et al [80], 2018	Some concerns	Low	Low	Some concerns	Low	Some concerns
Hoorelbeke and Koster [81], 2017	Low	Low	Low	Some concerns	Low	Some concerns
Jelinek et al [82], 2020	Some concerns	Low	Low	Some concerns	Low	Some concerns
Johansson et al [83], 2019	Low	Low	Low	Some concerns	Some concerns	Some concerns
Johansson et al [84], 2013	Low	Low	Low	Some concerns	Low	Some concerns
Johansson et al [85], 2012	Low	Low	Low	Some concerns	Low	Some concerns
Johansson et al [86], 2012	Low	Some concerns	Low	Some concerns	Low	Some concerns
Kessler et al [87], 2009	Low	Some concerns	Low	Some concerns	Low	Some concerns
Kivi et al [88], 2014	Low	High	High	Some concerns	Some concerns	High
Kladnitski et al [89], 2020	Low	Low	Low	Some concerns	Some concerns	Some concerns
Klein et al [90], 2017	Low	Low	Some concerns	Some concerns	Low	Some concerns
Kok et al [91], 2015	Some concerns	Low	Low	Some concerns	Low	Some concerns
Levesque et al [92], 2011	Some concerns	Low	Low	Some concerns	Some concerns	Some concerns
Levin et al [93], 2011	Some concerns	Low	Low	Some concerns	Some concerns	Some concerns
Lindegaard et al [94], 2021	Some concerns	Low	Low	Some concerns	Low	Some concerns
Lokman et al [95], 2017	Low	Low	Low	Some concerns	Low	Some concerns
Loughnan et al [96], 2019	Low	Low	Low	Some concerns	Low	Some concerns
Loughnan et al [97], 2019	Low	Low	Low	Some concerns	Low	Some concerns
Lu et al [98], 2021	Low	Low	Low	Some concerns	Low	Some concerns
Lüdtke et al [99], 2018	Some concerns	Low	Low	Some concerns	Low	Some concerns
Lukas, and Berking [100], 2021	Some concerns	Low	High	Some concerns	Low	High
McCloud et al [101], 2020	Low	Low	Low	Some concerns	Low	Some concerns
Meglic et al [102], 2010	High	Low	Low	Some concerns	Low	High
Meyer et al [103], 2015	Low	Low	Low	Some concerns	Low	Some concerns
Milgrom et al [104], 2016	Low	Low	Low	Some concerns	Low	Some concerns
Mira et al [105], 2017	Low	Low	Low	Some concerns	Low	Some concerns
Moberg et al [106], 2019	Low	Low	Low	Some concerns	Low	Some concerns
Monteiro et al [107], 2020	Low	Low	Some concerns	Some concerns	Low	Some concerns
Montero-Marin et al [108], 2016	Low	Low	Low	Some concerns	Low	Some concerns
Morgan et al [109], 2012	Low	Low	High	Some concerns	Low	High
Morgan et al [110], 2013	Low	Low	High	Some concerns	Low	High
Moritz et al [111], 2012	Low	Low	Low	Some concerns	Low	Some concerns
Mullin et al [112], 2015	Low	Low	Low	Some concerns	Low	Some concerns
Newby et al [113], 2013	Low	Low	Low	Some concerns	Low	Some concerns

Newby et al [114], 2014	Low	Some concerns	Low	Some concerns	Low	Some concerns
Noguchi et al [115], 2017	Low	Low	Low	Some concerns	Low	Some concerns
Nygren et al [116], 2019	Some concerns	Low	Low	Some concerns	Some concerns	Some concerns
O'Mahen et al [117], 2014	Low	Low	Low	Some concerns	Low	Some concerns
Oehler et al [118], 2020	Low	Low	Low	Some concerns	Low	Some concerns
Ofoegbu et al [119], 2020	Low	Some concerns	Low	Some concerns	Low	Some concerns
Pfeiffer et al [120], 2020	Low	Low	Low	Some concerns	Low	Some concerns
Phillips et al [121], 2014	Low	Low	Low	Low	Low	Low
Pictet et al [122], 2016	Some concerns	Low	Low	Some concerns	Low	Some concerns
Pots et al [123], 2016	Low	Low	Low	Some concerns	Low	Some concerns
Proudfoot et al [124], 2003	Low	Low	Low	Some concerns	Low	Some concerns
Proudfoot et al [125], 2004	Low	Low	Low	Some concerns	Low	Some concerns
Proudfoot et al [126], 2013	Low	Low	Low	Some concerns	Low	Some concerns
Reins et al [127], 2019	Low	Low	Low	Some concerns	Low	Some concerns
Richards et al [128], 2015	Low	Low	Some concerns	Some concerns	Low	Some concerns
Richards et al [129], 2020	Low	Low	Low	Some concerns	Low	Some concerns
Ritvo et al [130], 2021	Low	Low	Low	Some concerns	Low	Some concerns
Robichaud et al [131], 2020	Low	Low	Low	Some concerns	Low	Some concerns
Roepke et al [132], 2015	Low	Low	Low	Some concerns	Low	Some concerns
Rollman et al [133], 2018	Low	Low	Low	Low	Low	Low
Romero-Sanchiz et al [134], 2017	Low	Low	Low	Some concerns	Low	Some concerns
Rosso et al [135], 2016	Low	Low	Low	Low	Low	Low
Ruehlman and Karoly [136], 2021	Some concerns	High	High	Some concerns	Some concerns	High
Salamanca-Sanabria et al [137], 2020	Low	Low	Low	Some concerns	Low	Some concerns
Salisbury et al [138], 2016	Low	Low	Low	Some concerns	Low	Some concerns
Sandoval et al [139], 2017	Low	Low	Low	Some concerns	Low	Some concerns
Schure et al [140], 2019	Low	Low	Low	Some concerns	Low	Some concerns
Segal et al [141], 2020	Low	Low	Low	Some concerns	Low	Some concerns
Smith et al [142], 2017	Low	Some concerns	Low	Some concerns	Low	Some concerns
Sun et al [143], 2021	Low	Low	Low	Some concerns	Low	Some concerns
Terides et al [144], 2018	Low	Low	Low	Some concerns	Low	Some concerns
Titov et al [145], 2010	Low	Low	Low	Some concerns	Low	Some concerns
Titov et al [146], 2013	Low	Low	Low	Some concerns	Low	Some concerns
Tönnies et al [147], 2021	Low	Low	Low	Some concerns	Low	Some concerns
Tønning et al [148], 2021	Low	Low	Low	Low	Low	Low
Tulbure et al [149], 2018	High	High	Low	Some concerns	Low	High

Twomey et al [150], 2014	Some concerns	Low	Some concerns	Some concerns	Some concerns	Some concerns
Warmerdam et al [151], 2009	Low	Low	Low	Some concerns	Low	Some concerns
Yeung et al [152], 2018	Low	Some concerns	Low	Some concerns	Low	Some concerns
Zwerenz et al [153], 2017	Low	Low	Low	Some concerns	Low	Some concerns
