# Supplementary material for: Time-Dependent Changes in Depressive Symptoms Among Control Participants in Digital-Based Psychological Intervention Studies: Meta-analysis of Randomized Controlled Trials
Source: J Med Internet Res. 2023 Apr 12;25:e39029. doi: 10.2196/39029 (PMC10134030; doi:10.2196/39029)
Supplement: Multimedia Appendix 3 [file jmir_v25i1e39029_app3.pdf]

Multimedia Appendix 3.1.1

Forrest plot of immediate effects

Immediate effects

Study name	Outcome	Hedges's g	Standard error	Variance	Lower limit	Upper limit	Z-Value	p-Value	Total	Relative weight
Al-Alawi et al., 2021	PHQ-9	-0.942	0.217	0.047	-1.367	-0.516	-4.340	0.000	24	1.08
Beevers et al., 2017	Combined	-0.394	0.098	0.010	-0.586	-0.201	-4.003	0.000	91	1.48
Bohlmeijer et al., 2021a	CES-D	-0.411	0.109	0.012	-0.625	-0.197	-3.762	0.000	73	1.45
Bohlmeijer et al., 2021b	CES-D	-0.181	0.107	0.011	-0.391	0.029	-1.686	0.092	71	1.45
Browning et al., 2012a	Combined	0.022	0.229	0.052	-0.427	0.471	0.095	0.924	14	1.04
Browning et al., 2012b	Combined	-0.527	0.238	0.057	-0.993	-0.060	-2.213	0.027	15	1.01
Buntrock et al., 2015	CES-D	-0.310	0.065	0.004	-0.437	-0.183	-4.793	0.000	204	1.56
Calkins et al., 2015	BDI-II	-0.078	0.179	0.032	-0.429	0.273	-0.437	0.662	24	1.21
Clarke et al., 2005	CES-D	-0.322	0.092	0.009	-0.502	-0.141	-3.489	0.000	100	1.49
Clarke et al., 2009	PHQ-8	-0.031	0.102	0.010	-0.231	0.169	-0.303	0.762	77	1.47
Dainer-Best et al., 2018	CES-D	-0.932	0.091	0.008	-1.111	-0.753	-10.205	0.000	140	1.50
Day, McGrath & Wojtowicz, 2013	DASS-21-D	-0.517	0.164	0.027	-0.839	-0.195	-3.145	0.002	33	1.26
De Graaf et al., 2011	BDI-II	-0.623	0.097	0.009	-0.813	-0.433	-6.430	0.000	103	1.48
Flygare et al., 2020	Combined	-0.665	0.144	0.021	-0.948	-0.381	-4.602	0.000	47	1.33
Fonseca et al., 2020	EPDS	-0.292	0.094	0.009	-0.475	-0.108	-3.114	0.002	96	1.49
Geraedts et al., 2014	CES-D	-0.932	0.101	0.010	-1.130	-0.735	-9.260	0.000	115	1.47
Gili et al., 2020	PHQ-9	-0.378	0.123	0.015	-0.618	-0.138	-3.085	0.002	57	1.40
Hallford et al., 2021	PHQ-9	-0.634	0.090	0.008	-0.811	-0.458	-7.037	0.000	120	1.50
Harrer et al., 2021	CES-D	-0.332	0.092	0.009	-0.513	-0.151	-3.594	0.000	100	1.49
Hatcher et al., 2018	PHQ-9	-0.919	0.204	0.041	-1.318	-0.520	-4.512	0.000	27	1.13
Heim et al., 2021	PHQ-9	-0.872	0.125	0.016	-1.117	-0.626	-6.959	0.000	71	1.40
Hirsch et al., 2018	PHQ-9	-0.371	0.149	0.022	-0.663	-0.079	-2.491	0.013	38	1.32
Hobfoll et al., 2016	CESD-10	0.007	0.093	0.009	-0.174	0.189	0.081	0.936	94	1.49
Hoifodt et al., 2013	BDI-II	-0.462	0.128	0.016	-0.713	-0.211	-3.611	0.000	54	1.39
Hoorelbeke & Koster, 2017	BDI-II	0.235	0.154	0.024	-0.067	0.537	1.527	0.127	34	1.30
Jelinek et al., 2020a	PHQ-9	-0.193	0.158	0.025	-0.502	0.116	-1.226	0.220	32	1.29
Jelinek et al., 2020b	PHQ-9	-0.278	0.153	0.023	-0.577	0.021	-1.822	0.068	35	1.30
Johansson et al., 2019	Combined	-0.114	0.170	0.029	-0.447	0.219	-0.672	0.501	27	1.24
Kessler et al., 2009	BDI-II	-0.943	0.089	0.008	-1.118	-0.769	-10.581	0.000	148	1.50
Kladnitski et al., 2020	PHQ-9	-0.251	0.144	0.021	-0.534	0.032	-1.739	0.082	39	1.33
Kok et al., 2015	IDS-SR	0.421	0.088	0.008	0.247	0.594	4.762	0.000	113	1.50
Levin et al., 2011	Combined	-0.382	0.098	0.010	-0.574	-0.191	-3.917	0.000	91	1.48
Lindegaard et al., 2019	Combined	-0.241	0.180	0.032	-0.594	0.111	-1.344	0.179	25	1.21
Lindegaard et al., 2021	PHQ-9	-0.085	0.164	0.027	-0.406	0.237	-0.516	0.606	29	1.27
Loughnan et al., 2019a	Combined	-0.605	0.152	0.023	-0.903	-0.307	-3.976	0.000	41	1.31
Loughnan et al., 2019b	Combined	-0.418	0.126	0.016	-0.665	-0.172	-3.325	0.001	55	1.39
Lu et al., 2021	PHQ-9	-0.139	0.183	0.034	-0.498	0.220	-0.757	0.449	23	1.20
Ludtke et al., 2018	PHQ-9	-0.328	0.112	0.013	-0.548	-0.108	-2.918	0.004	67	1.44
Lukas et al., 2021	Combined	-0.169	0.147	0.022	-0.457	0.119	-1.151	0.250	37	1.32
McCloud et al., 2020	HADS	-0.344	0.101	0.010	-0.541	-0.146	-3.410	0.001	84	1.47
Moberg, Niles & Beermann, 2019	Combined	-0.090	0.058	0.003	-0.203	0.023	-1.562	0.118	247	1.57
Monteiro et al., 2020	EPDS	-0.156	0.068	0.005	-0.290	-0.022	-2.288	0.022	176	1.55
Morgan, Jorm & Mackinnon, 2012	PHQ-9	-0.502	0.037	0.001	-0.575	-0.428	-13.436	0.000	661	1.61
Morgan, Jorm & Mackinnon, 2013	PHQ-9	-0.855	0.036	0.001	-0.925	-0.785	-23.900	0.000	874	1.61
Moritz et al., 2012	BDI-II	-0.392	0.091	0.008	-0.571	-0.214	-4.306	0.000	105	1.50
Mullin et al., 2015	PHQ-9	-0.214	0.185	0.034	-0.575	0.148	-1.158	0.247	23	1.19
Newby et al., 2014	BDI-II	-0.152	0.196	0.038	-0.536	0.231	-0.778	0.436	20	1.16
Noguchi et al., 2017	Combined	-0.054	0.050	0.003	-0.153	0.044	-1.086	0.277	325	1.59
Nygren et al., 2019	Combined	-0.241	0.180	0.032	-0.594	0.111	-1.344	0.179	25	1.21
Oehler et al., 2020	Combined	-0.367	0.071	0.005	-0.506	-0.228	-5.193	0.000	174	1.55
Philips et al., 2014	PHQ-9	-0.755	0.057	0.003	-0.868	-0.642	-13.141	0.000	318	1.57
Pictet, Jermann & Ceschi, 2016a	BDI-II	-0.378	0.157	0.025	-0.686	-0.070	-2.403	0.016	34	1.29
Pictet, Jermann & Ceschi, 2016b	BDI-II	-0.105	0.154	0.024	-0.407	0.198	-0.679	0.497	33	1.30
Proudfoot et al., 2004	BDI-II	-0.871	0.098	0.010	-1.064	-0.679	-8.866	0.000	116	1.48
Proudfoot et al., 2013a	DASS-21-D	-0.129	0.058	0.003	-0.242	-0.015	-2.226	0.026	246	1.57
Proudfoot et al., 2013b	DASS-21-D	-0.001	0.060	0.004	-0.118	0.116	-0.018	0.986	228	1.57
Reins et al., 2019	HRSD	-0.584	0.119	0.014	-0.818	-0.350	-4.891	0.000	66	1.41
Richards et al., 2015	BDI-II	-0.065	0.094	0.009	-0.249	0.118	-0.698	0.485	92	1.49
Richards et al., 2020	PHQ-9	-0.477	0.087	0.008	-0.647	-0.307	-5.497	0.000	120	1.51
Robichaud et al., 2020	PHQ-9	-0.581	0.172	0.030	-0.919	-0.244	-3.379	0.001	31	1.24
Roepke et al., 2015	CES-D	-0.502	0.099	0.010	-0.695	-0.308	-5.071	0.000	93	1.48
Ruehlman et al., 2021	PHQ-8	-0.388	0.186	0.035	-0.752	-0.024	-2.089	0.037	24	1.19
Salamanca-Sanabria et al., 2020	PHQ-9	-0.161	0.087	0.008	-0.332	0.011	-1.836	0.066	107	1.51
Sandoval et al., 2017	BDI-II	0.115	0.195	0.038	-0.268	0.497	0.589	0.556	20	1.16
Schure et al., 2019	PHQ-9	-0.768	0.081	0.007	-0.926	-0.610	-9.522	0.000	162	1.52
Sun et al., 2021	EPDS	-0.184	0.099	0.010	-0.378	0.009	-1.867	0.062	84	1.48
Terides et al., 2018	PHQ-9	-0.237	0.106	0.011	-0.444	-0.030	-2.246	0.025	74	1.46
Titov et al., 2010	Combined	-0.049	0.141	0.020	-0.324	0.227	-0.346	0.730	40	1.35
Titov et al., 2013	PHQ-9	-0.006	0.125	0.016	-0.251	0.239	-0.049	0.961	51	1.40
Twomey et al., 2014	DASS-21-D	0.000	0.144	0.021	-0.282	0.282	0.000	1.000	38	1.33
Warmerdam et al., 2009	CES-D	-0.670	0.107	0.011	-0.879	-0.461	-6.281	0.000	87	1.45
Yeung et al., 2018	CES-D	-0.858	0.169	0.029	-1.190	-0.526	-5.069	0.000	38	1.25
		-0.358	0.039	0.002	-0.434	-0.281	-9.172	0.000		

Statistics for each study

Hedges's g and 95% CI

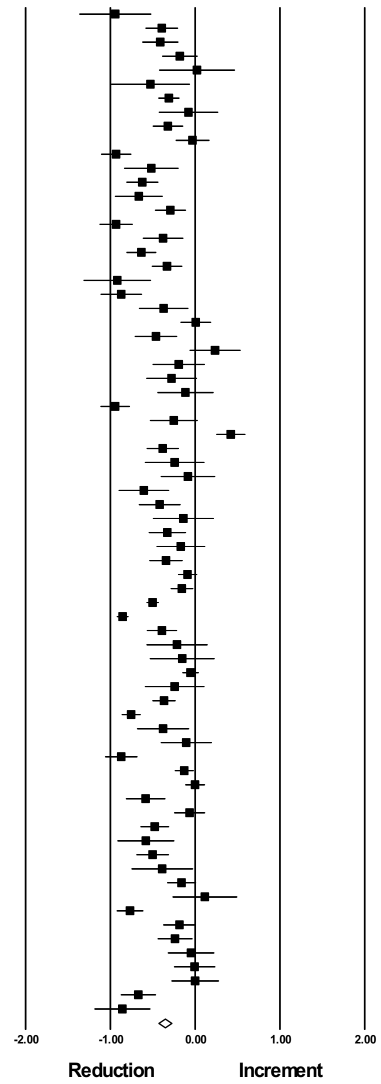

Meta Analysis

Multimedia Appendix 3.1.2
Forrest plot of immediate effects by control type

Immediate effects by control type

Group by Control_type	Study name	Outcome	Statistics for each study: Hedges's g	Standard error	Variance	Lower limit	Upper limit	Z-Value	p-Value	Total	Relative weight
Active	Bohlmeijer et al., 2021a	CES-D	-0.411	0.109	0.012	-0.625	-0.197	-3.762	0.000	73	26.35
Active	Hoorelbeke & Koster, 2017	BDI-II	0.235	0.154	0.024	-0.067	0.537	1.527	0.127	34	22.15
Active	Jelinek et al., 2020a	PHQ-9	-0.193	0.158	0.025	-0.502	0.116	-1.226	0.220	32	21.81
Active	Oehler et al., 2020	Combined	-0.367	0.071	0.005	-0.506	-0.228	-5.193	0.000	174	29.68
Active			-0.207	0.128	0.016	-0.458	0.043	-1.623	0.105		
CAU	Beevers et al., 2017	Combined	-0.394	0.098	0.010	-0.586	-0.201	-4.003	0.000	91	4.59
CAU	De Graaf et al., 2011	BDI-II	-0.623	0.097	0.009	-0.813	-0.433	-6.430	0.000	103	4.61
CAU	Fonseca et al., 2020	EPDS	-0.292	0.094	0.009	-0.475	-0.108	-3.114	0.002	96	4.64
CAU	Geraedts et al., 2014	CES-D	-0.932	0.101	0.010	-1.130	-0.735	-9.260	0.000	115	4.57
CAU	Gili et al., 2020	PHQ-9	-0.378	0.123	0.015	-0.618	-0.138	-3.085	0.002	57	4.36
CAU	Hoifodt et al., 2013	BDI-II	-0.462	0.128	0.016	-0.713	-0.211	-3.611	0.000	54	4.30
CAU	Jelinek et al., 2020b	PHQ-9	-0.278	0.153	0.023	-0.577	0.021	-1.822	0.068	35	4.04
CAU	Johansson et al., 2019	Combined	-0.114	0.170	0.029	-0.447	0.219	-0.672	0.501	27	3.85
CAU	Kladnitski et al., 2020	PHQ-9	-0.251	0.144	0.021	-0.534	0.032	-1.739	0.082	39	4.13
CAU	Kok et al., 2015	IDS-SR	0.421	0.088	0.008	0.247	0.594	4.762	0.000	113	4.68
CAU	Levin et al., 2011	Combined	-0.382	0.098	0.010	-0.574	-0.191	-3.917	0.000	91	4.60
CAU	Loughnan et al., 2019a	Combined	-0.605	0.152	0.023	-0.903	-0.307	-3.976	0.000	41	4.04
CAU	Loughnan et al., 2019b	Combined	-0.418	0.126	0.016	-0.665	-0.172	-3.325	0.001	55	4.33
CAU	Lu et al., 2021	PHQ-9	-0.139	0.183	0.034	-0.498	0.220	-0.757	0.449	23	3.70
CAU	Ludtke et al., 2018	PHQ-9	-0.328	0.112	0.013	-0.548	-0.108	-2.918	0.004	67	4.46
CAU	McCloud et al., 2020	HADS	-0.344	0.101	0.010	-0.541	-0.146	-3.410	0.001	84	4.57
CAU	Monteiro et al., 2020	EPDS	-0.156	0.068	0.005	-0.290	-0.022	-2.288	0.022	176	4.84
CAU	Proudfoot et al., 2004	BDI-II	-0.871	0.098	0.010	-1.064	-0.679	-8.866	0.000	116	4.59
CAU	Richards et al., 2020	PHQ-9	-0.477	0.087	0.008	-0.647	-0.307	-5.497	0.000	120	4.69
CAU	Robichaud et al., 2020	PHQ-9	-0.581	0.172	0.030	-0.919	-0.244	-3.379	0.001	31	3.83
CAU	Roepke et al., 2015	CES-D	-0.502	0.099	0.010	-0.695	-0.308	-5.071	0.000	93	4.59
CAU	Twomey et al., 2014	DASS-21-D	0.000	0.144	0.021	-0.282	0.282	0.000	1.000	38	4.13
CAU	Yeung et al., 2018	CES-D	-0.858	0.169	0.029	-1.190	-0.526	-5.069	0.000	38	3.86
CAU			-0.390	0.068	0.005	-0.522	-0.257	-5.763	0.000		
Information	Al-Alawi et al., 2021	PHQ-9	-0.942	0.217	0.047	-1.367	-0.516	-4.340	0.000	24	6.11
Information	Buntrock et al., 2015	CES-D	-0.310	0.065	0.004	-0.437	-0.183	-4.793	0.000	204	9.04
Information	Clarke et al., 2005	CES-D	-0.322	0.092	0.009	-0.502	-0.141	-3.489	0.000	100	8.63
Information	Clarke et al., 2009	PHQ-8	-0.031	0.102	0.010	-0.231	0.169	-0.303	0.762	77	8.45
Information	Harrer et al., 2021	CES-D	-0.332	0.092	0.009	-0.513	-0.151	-3.594	0.000	100	8.62
Information	Hatcher et al., 2018	PHQ-9	-0.919	0.204	0.041	-1.318	-0.520	-4.512	0.000	27	6.39
Information	Heim et al., 2021	PHQ-9	-0.872	0.125	0.016	-1.117	-0.626	-6.959	0.000	71	8.01
Information	Morgan, Jorm & Mackinnon, 2012	PHQ-9	-0.502	0.037	0.001	-0.575	-0.428	-13.436	0.000	661	9.33
Information	Morgan, Jorm & Mackinnon, 2013	PHQ-9	-0.855	0.036	0.001	-0.925	-0.785	-23.900	0.000	874	9.34
Information	Proudfoot et al., 2013a	DASS-21-D	-0.129	0.058	0.003	-0.242	-0.015	-2.226	0.026	246	9.13
Information	Reins et al., 2019	HRSD	-0.584	0.119	0.014	-0.818	-0.350	-4.891	0.000	66	8.13
Information	Schure et al., 2019	PHQ-9	-0.768	0.081	0.007	-0.926	-0.610	-9.522	0.000	162	8.81
Information			-0.527	0.090	0.008	-0.703	-0.350	-5.852	0.000		
Sham	Browning et al., 2012a	Combined	0.022	0.229	0.052	-0.427	0.471	0.095	0.924	14	8.86
Sham	Browning et al., 2012b	Combined	-0.527	0.238	0.057	-0.993	-0.060	-2.213	0.027	15	8.58
Sham	Calkins et al., 2015	BDI-II	-0.078	0.179	0.032	-0.429	0.273	-0.437	0.662	24	10.50
Sham	Dainer-Best et al., 2018	CES-D	-0.932	0.091	0.008	-1.111	-0.753	-10.205	0.000	140	13.40
Sham	Flygare et al., 2020	Combined	-0.665	0.144	0.021	-0.948	-0.381	-4.602	0.000	47	11.70
Sham	Hirsch et al., 2018	PHQ-9	-0.371	0.149	0.022	-0.663	-0.079	-2.491	0.013	38	11.54
Sham	Newby et al., 2014	BDI-II	-0.152	0.196	0.038	-0.536	0.231	-0.778	0.436	20	9.94
Sham	Philips et al., 2014	PHQ-9	-0.755	0.057	0.003	-0.868	-0.642	-13.141	0.000	318	14.23
Sham	Pictet, Jermann & Ceschi, 2016a	BDI-II	-0.378	0.157	0.025	-0.686	-0.070	-2.403	0.016	34	11.25
Sham			-0.462	0.107	0.012	-0.673	-0.251	-4.301	0.000		
WL	Bohlmeijer et al., 2021b	CES-D	-0.181	0.107	0.011	-0.391	0.029	-1.686	0.092	71	4.60
WL	Day, McGrath & Wojtowicz, 2013	DASS-21-D	-0.517	0.164	0.027	-0.839	-0.195	-3.145	0.002	33	3.80
WL	Hallford et al., 2021	PHQ-9	-0.634	0.090	0.008	-0.811	-0.458	-7.037	0.000	120	4.82
WL	Hobfoll et al., 2016	CESD-10	0.007	0.093	0.009	-0.174	0.189	0.081	0.936	94	4.79
WL	Kessler et al., 2009	BDI-II	-0.943	0.089	0.008	-1.118	-0.769	-10.581	0.000	148	4.84
WL	Lindegaard et al., 2019	Combined	-0.241	0.180	0.032	-0.594	0.111	-1.344	0.179	25	3.59
WL	Lindegaard et al., 2021	PHQ-9	-0.085	0.164	0.027	-0.406	0.237	-0.516	0.606	29	3.81
WL	Lukas et al., 2021	Combined	-0.169	0.147	0.022	-0.457	0.119	-1.151	0.250	37	4.05
WL	Moberg, Niles & Beermann, 2019	Combined	-0.090	0.058	0.003	-0.203	0.023	-1.562	0.118	247	5.18
WL	Moritz et al., 2012	BDI-II	-0.392	0.091	0.008	-0.571	-0.214	-4.306	0.000	105	4.81
WL	Mullin et al., 2015	PHQ-9	-0.214	0.185	0.034	-0.575	0.148	-1.158	0.247	23	3.52
WL	Noguchi et al., 2017	Combined	-0.054	0.050	0.003	-0.153	0.044	-1.086	0.277	325	5.24
WL	Nygren et al., 2019	Combined	-0.241	0.180	0.032	-0.594	0.111	-1.344	0.179	25	3.59
WL	Pictet, Jermann & Ceschi, 2016b	BDI-II	-0.105	0.154	0.024	-0.407	0.198	-0.679	0.497	33	3.94
WL	Proudfoot et al., 2013b	DASS-21-D	-0.001	0.060	0.004	-0.118	0.116	-0.018	0.986	228	5.16
WL	Richards et al., 2015	BDI-II	-0.065	0.094	0.009	-0.249	0.118	-0.698	0.485	92	4.78
WL	Ruehlman et al., 2021	PHQ-8	-0.388	0.186	0.035	-0.752	-0.024	-2.089	0.037	24	3.51
WL	Salamanca-Sanabria et al., 2020	PHQ-9	-0.161	0.087	0.008	-0.332	0.011	-1.836	0.066	107	4.86
WL	Sandoval et al., 2017	BDI-II	0.115	0.195	0.038	-0.268	0.497	0.589	0.556	20	3.38
WL	Terides et al., 2018	PHQ-9	-0.237	0.106	0.011	-0.444	-0.030	-2.246	0.025	74	4.62
WL	Titov et al., 2010	Combined	-0.049	0.141	0.020	-0.324	0.227	-0.346	0.730	40	4.14
WL	Titov et al., 2013	PHQ-9	-0.006	0.125	0.016	-0.251	0.239	-0.049	0.961	51	4.36
WL	Warmerdam et al., 2009	CES-D	-0.670	0.107	0.011	-0.879	-0.461	-6.281	0.000	87	4.61
WL			-0.234	0.058	0.003	-0.348	-0.120	-4.021	0.000		
Overall			-0.346	0.036	0.001	-0.416	-0.277	-9.709	0.000		

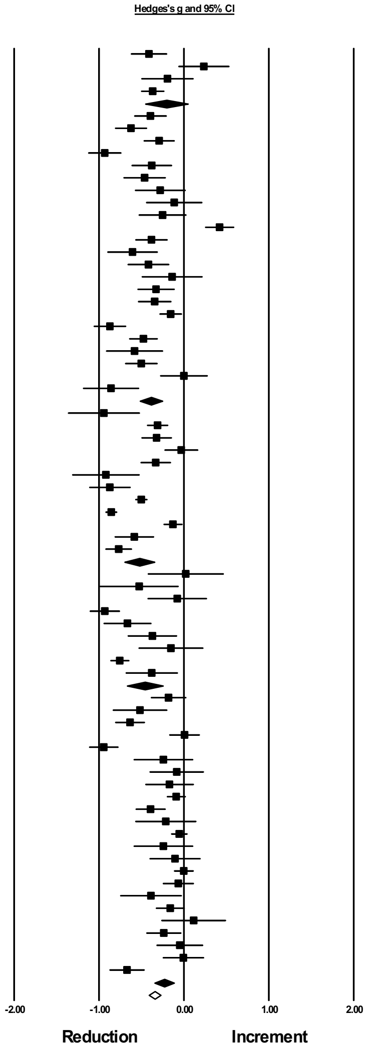

Meta Analysis

Multimedia Appendix 3.1.3
Forrest plot of immediate effects by severity

Immediate effects by severity

Group by Severity	Studyname	Outcome	Hedges's g	Standard error	Variance	Lower limit	Upper limit	Z-Value	p-Value	Total	Relative weight
Mild	Bohlmeijer et al., 2021a	CES-D	-0.411	0.109	0.012	-0.625	-0.197	-3.762	0.000	73	9.57
Mild	Bohlmeijer et al., 2021b	CES-D	-0.181	0.107	0.011	-0.391	0.029	-1.686	0.092	71	9.63
Mild	Browning et al., 2012a	Combined	0.022	0.229	0.052	-0.427	0.471	0.095	0.924	14	6.13
Mild	Browning et al., 2012b	Combined	-0.527	0.238	0.057	-0.993	-0.060	-2.213	0.027	15	5.91
Mild	Fonseca et al., 2020	EPDS	-0.292	0.094	0.009	-0.475	-0.108	-3.114	0.002	96	10.01
Mild	Hobfoll et al., 2016	CESD-10	0.007	0.093	0.009	-0.174	0.189	0.081	0.936	94	10.04
Mild	Hoorelbeke & Koster, 2017	BDI-II	0.235	0.154	0.024	-0.067	0.537	1.527	0.127	34	8.23
Mild	Kok et al., 2015	IDS-SR	0.421	0.088	0.008	0.247	0.594	4.762	0.000	113	10.15
Mild	McCloud et al., 2020	HADS	-0.344	0.101	0.010	-0.541	-0.146	-3.410	0.001	84	9.81
Mild	Monteiro et al., 2020	EPDS	-0.156	0.068	0.005	-0.290	-0.022	-2.288	0.022	176	10.64
Mild	Sun et al., 2021	EPDS	-0.184	0.099	0.010	-0.378	0.009	-1.867	0.062	84	9.87
Mild			-0.122	0.083	0.007	-0.285	0.041	-1.461	0.144		
Moderate	Calkins et al., 2015	BDI-II	-0.078	0.179	0.032	-0.429	0.273	-0.437	0.662	24	2.18
Moderate	Clarke et al., 2009	PHQ-8	-0.031	0.102	0.010	-0.231	0.169	-0.303	0.762	77	2.82
Moderate	Day, McGrath & Wojtowicz, 2013	DASS-21-D	-0.517	0.164	0.027	-0.839	-0.195	-3.145	0.002	33	2.30
Moderate	De Graaf et al., 2011	BDI-II	-0.623	0.097	0.009	-0.813	-0.433	-6.430	0.000	103	2.87
Moderate	Flygare et al., 2020	Combined	-0.665	0.144	0.021	-0.948	-0.381	-4.602	0.000	47	2.47
Moderate	Gili et al., 2020	PHQ-9	-0.378	0.123	0.015	-0.618	-0.138	-3.085	0.002	57	2.66
Moderate	Harrer et al., 2021	CES-D	-0.332	0.092	0.009	-0.513	-0.151	-3.594	0.000	100	2.90
Moderate	Hoifodt et al., 2013	BDI-II	-0.462	0.128	0.016	-0.713	-0.211	-3.611	0.000	54	2.61
Moderate	Jelinek et al., 2020a	PHQ-9	-0.193	0.158	0.025	-0.502	0.116	-1.226	0.220	32	2.36
Moderate	Jelinek et al., 2020b	PHQ-9	-0.278	0.153	0.023	-0.577	0.021	-1.822	0.068	35	2.40
Moderate	Johansson et al., 2019	Combined	-0.114	0.170	0.029	-0.447	0.219	-0.672	0.501	27	2.26
Moderate	Kladnitski et al., 2020	PHQ-9	-0.251	0.144	0.021	-0.534	0.032	-1.739	0.082	39	2.47
Moderate	Loughnan et al., 2019a	Combined	-0.605	0.152	0.023	-0.903	-0.307	-3.976	0.000	41	2.41
Moderate	Loughnan et al., 2019b	Combined	-0.418	0.126	0.016	-0.665	-0.172	-3.325	0.001	55	2.63
Moderate	Lu et al., 2021	PHQ-9	-0.139	0.183	0.034	-0.498	0.220	-0.757	0.449	23	2.15
Moderate	Ludtke et al., 2018	PHQ-9	-0.328	0.112	0.013	-0.548	-0.108	-2.918	0.004	67	2.74
Moderate	Morgan, Jorm & Mackinnon, 2012	PHQ-9	-0.502	0.037	0.001	-0.575	-0.428	-13.436	0.000	661	3.22
Moderate	Mullin et al., 2015	PHQ-9	-0.214	0.185	0.034	-0.575	0.148	-1.158	0.247	23	2.14
Moderate	Newby et al., 2014	BDI-II	-0.152	0.196	0.038	-0.536	0.231	-0.778	0.436	20	2.05
Moderate	Noguchi et al., 2017	Combined	-0.054	0.050	0.003	-0.153	0.044	-1.086	0.277	325	3.17
Moderate	Oehler et al., 2020	Combined	-0.367	0.071	0.005	-0.506	-0.228	-5.193	0.000	174	3.05
Moderate	Philips et al., 2014	PHQ-9	-0.755	0.057	0.003	-0.868	-0.642	-13.141	0.000	318	3.13
Moderate	Pictet, Jermann & Ceschi, 2016a	BDI-II	-0.378	0.157	0.025	-0.686	-0.070	-2.403	0.016	34	2.36
Moderate	Pictet, Jermann & Ceschi, 2016b	BDI-II	-0.105	0.154	0.024	-0.407	0.198	-0.679	0.497	33	2.39
Moderate	Proudfoot et al., 2004	BDI-II	-0.871	0.098	0.010	-1.064	-0.679	-8.866	0.000	116	2.86
Moderate	Proudfoot et al., 2013a	DASS-21-D	-0.129	0.058	0.003	-0.242	-0.015	-2.226	0.026	246	3.13
Moderate	Proudfoot et al., 2013b	DASS-21-D	-0.001	0.060	0.004	-0.118	0.116	-0.018	0.986	228	3.12
Moderate	Reins et al., 2019	HRSD	-0.584	0.119	0.014	-0.818	-0.350	-4.891	0.000	66	2.68
Moderate	Richards et al., 2015	BDI-II	-0.065	0.094	0.009	-0.249	0.118	-0.698	0.485	92	2.89
Moderate	Richards et al., 2020	PHQ-9	-0.477	0.087	0.008	-0.647	-0.307	-5.497	0.000	120	2.94
Moderate	Robichaud et al., 2020	PHQ-9	-0.581	0.172	0.030	-0.919	-0.244	-3.379	0.001	31	2.24
Moderate	Salamanca-Sanabria et al., 2020	PHQ-9	-0.161	0.087	0.008	-0.332	0.011	-1.836	0.066	107	2.94
Moderate	Sandoval et al., 2017	BDI-II	0.115	0.195	0.038	-0.268	0.497	0.589	0.556	20	2.05
Moderate	Schure et al., 2019	PHQ-9	-0.768	0.081	0.007	-0.926	-0.610	-9.522	0.000	162	2.99
Moderate	Terides et al., 2018	PHQ-9	-0.237	0.106	0.011	-0.444	-0.030	-2.246	0.025	74	2.80
Moderate	Titov et al., 2010	Combined	-0.049	0.141	0.020	-0.324	0.227	-0.346	0.730	40	2.51
Moderate	Titov et al., 2013	PHQ-9	-0.006	0.125	0.016	-0.251	0.239	-0.049	0.961	51	2.64
Moderate	Twomey et al., 2014	DASS-21-D	0.000	0.144	0.021	-0.282	0.282	0.000	1.000	38	2.48
Moderate			-0.317	0.045	0.002	-0.406	-0.228	-6.967	0.000		
Severe	Al-Alawi et al., 2021	PHQ-9	-0.942	0.217	0.047	-1.367	-0.516	-4.340	0.000	24	3.55
Severe	Buntrock et al., 2015	CES-D	-0.310	0.065	0.004	-0.437	-0.183	-4.793	0.000	204	5.55
Severe	Clarke et al., 2005	CES-D	-0.322	0.092	0.009	-0.502	-0.141	-3.489	0.000	100	5.26
Severe	Dainer-Best et al., 2018	CES-D	-0.932	0.091	0.008	-1.111	-0.753	-10.205	0.000	140	5.27
Severe	Geraedts et al., 2014	CES-D	-0.932	0.101	0.010	-1.130	-0.735	-9.260	0.000	115	5.15
Severe	Hallford et al., 2021	PHQ-9	-0.634	0.090	0.008	-0.811	-0.458	-7.037	0.000	120	5.28
Severe	Hatcher et al., 2018	PHQ-9	-0.919	0.204	0.041	-1.318	-0.520	-4.512	0.000	27	3.73
Severe	Heim et al., 2021	PHQ-9	-0.872	0.125	0.016	-1.117	-0.626	-6.959	0.000	71	4.83
Severe	Hirsch et al., 2018	PHQ-9	-0.371	0.149	0.022	-0.663	-0.079	-2.491	0.013	38	4.49
Severe	Kessler et al., 2009	BDI-II	-0.943	0.089	0.008	-1.118	-0.769	-10.581	0.000	148	5.29
Severe	Levin et al., 2011	CES-D	-0.347	0.097	0.009	-0.537	-0.157	-3.581	0.000	91	5.20
Severe	Lindegaard et al., 2019	Combined	-0.241	0.180	0.032	-0.594	0.111	-1.344	0.179	25	4.06
Severe	Lindegaard et al., 2021	PHQ-9	-0.085	0.164	0.027	-0.406	0.237	-0.516	0.606	29	4.28
Severe	Lukas et al., 2021	Combined	-0.169	0.147	0.022	-0.457	0.119	-1.151	0.250	37	4.52
Severe	Morgan, Jorm & Mackinnon, 2013	PHQ-9	-0.855	0.036	0.001	-0.925	-0.785	-23.900	0.000	874	5.77
Severe	Moritz et al., 2012	BDI-II	-0.392	0.091	0.008	-0.571	-0.214	-4.306	0.000	105	5.27
Severe	Nygren et al., 2019	Combined	-0.241	0.180	0.032	-0.594	0.111	-1.344	0.179	25	4.06
Severe	Roepke et al., 2015	CES-D	-0.502	0.099	0.010	-0.695	-0.308	-5.071	0.000	93	5.17
Severe	Ruehlman et al., 2021	PHQ-8	-0.388	0.186	0.035	-0.752	-0.024	-2.089	0.037	24	3.97
Severe	Warmerdam et al., 2009	CES-D	-0.670	0.107	0.011	-0.879	-0.461	-6.281	0.000	87	5.08
Severe	Yeung et al., 2018	CES-D	-0.858	0.169	0.029	-1.190	-0.526	-5.069	0.000	38	4.21
Severe			-0.572	0.065	0.004	-0.699	-0.444	-8.780	0.000		
Overall			-0.354	0.034	0.001	-0.420	-0.287	-10.398	0.000		

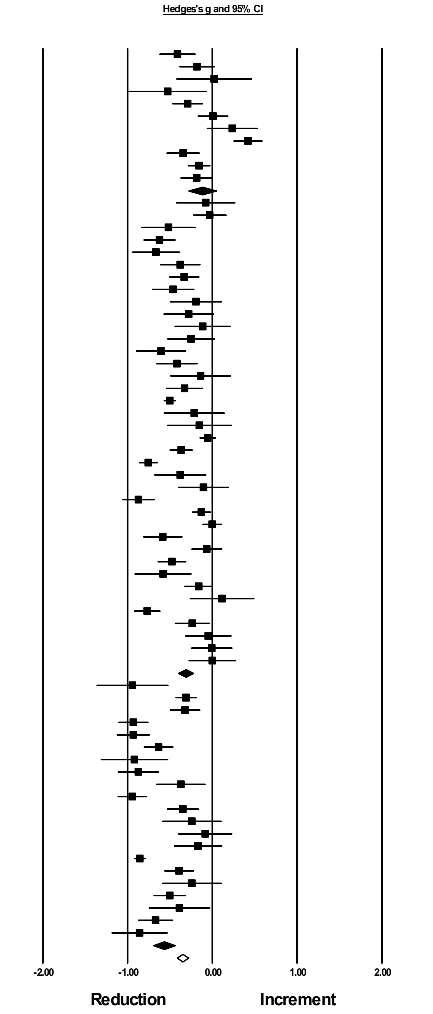

Meta Analysis

Multimedia Appendix 3.2.1
Forrest plot of short-term effects

Short-term effects

Study name	Outcome	Hedges's g	Standard error	Variance	Lower limit	Upper limit	Z-Value	p-Value	Total	Relative weight
Andersson et al., 2005	Combined	-0.256	0.130	0.017	-0.510	-0.001	-1.970	0.049	49	1.50
Berger et al., 2018	BDI-II	-0.385	0.135	0.018	-0.650	-0.121	-2.858	0.004	47	1.48
Birney et al., 2016	PHQ-9	-0.895	0.087	0.008	-1.066	-0.724	-10.269	0.000	150	1.60
Tulbure et al., 2018	BDI-II	-0.888	0.205	0.042	-1.290	-0.486	-4.328	0.000	26	1.26
Bohlmeijer et al., 2021a	CES-D	-0.696	0.117	0.014	-0.925	-0.466	-5.940	0.000	73	1.53
Bohlmeijer et al., 2021b	CES-D	-0.175	0.107	0.011	-0.385	0.035	-1.637	0.102	71	1.56
Boschloo et al., 2019	PHQ-9	-0.256	0.046	0.002	-0.346	-0.166	-5.564	0.000	399	1.68
Braun et al., 2021	QIDS-SR	-0.666	0.077	0.006	-0.816	-0.516	-8.681	0.000	169	1.63
Choi et al., 2012	BDI-II	0.055	0.161	0.026	-0.260	0.371	0.344	0.731	30	1.40
Clarke et al., 2005	CES-D	-0.409	0.094	0.009	-0.592	-0.225	-4.365	0.000	100	1.59
Clarke et al., 2009	PHQ-8	-0.046	0.102	0.010	-0.247	0.154	-0.454	0.650	77	1.57
De Graaf et al., 2011	BDI-II	-0.848	0.103	0.011	-1.051	-0.645	-8.194	0.000	103	1.57
Eriksson et al., 2017	BDI-II	-1.320	0.201	0.041	-1.715	-0.925	-6.555	0.000	37	1.28
Gilbody et al., 2015	PHQ-9	-1.312	0.080	0.006	-1.468	-1.155	-16.446	0.000	239	1.62
Hallford et al., 2021	PHQ-9	-0.886	0.097	0.009	-1.077	-0.696	-9.126	0.000	120	1.58
Hallgren et al., 2016	MADRS-S	-0.837	0.059	0.004	-0.954	-0.721	-14.077	0.000	312	1.66
Hange et al., 2017	MADRS-S	-1.369	0.223	0.050	-1.807	-0.931	-6.127	0.000	31	1.21
Harrer et al., 2021	CES-D	-0.595	0.098	0.010	-0.786	-0.404	-6.095	0.000	100	1.58
Hatcher et al., 2018	PHQ-9	-1.069	0.214	0.046	-1.489	-0.649	-4.985	0.000	27	1.23
Heim et al., 2021	PHQ-9	-0.782	0.122	0.015	-1.021	-0.543	-6.422	0.000	71	1.52
Hobfoll et al., 2016	CESD-10	0.086	0.093	0.009	-0.096	0.268	0.926	0.355	94	1.59
Holländare et al., 2011	Combined	-0.339	0.141	0.020	-0.615	-0.062	-2.399	0.016	42	1.46
Holst et al., 2018	BDI-II	-1.320	0.201	0.041	-1.715	-0.925	-6.555	0.000	37	1.28
Hoorelbeke & Koster, 2017	BDI-II	0.288	0.155	0.024	-0.016	0.592	1.859	0.063	34	1.42
Johansson et al., 2012a	BDI-II	-0.878	0.155	0.024	-1.182	-0.573	-5.654	0.000	46	1.42
Johansson et al., 2012b	Combined	-0.517	0.146	0.021	-0.804	-0.230	-3.532	0.000	42	1.45
Johansson et al., 2013	PHQ-9	-0.671	0.140	0.020	-0.945	-0.397	-4.793	0.000	50	1.47
Kivi et al., 2014	Combined	-1.011	0.185	0.034	-1.375	-0.648	-5.453	0.000	35	1.33
Kladnitski et al., 2020	PHQ-9	-0.143	0.143	0.020	-0.423	0.137	-1.001	0.317	39	1.46
Klein et al., 2017	Combined	-0.257	0.041	0.002	-0.338	-0.177	-6.282	0.000	504	1.68
Kok et al., 2015	IDS-SR	0.479	0.089	0.008	0.304	0.654	5.361	0.000	113	1.60
Lokman et al., 2017	IDS-SR	-0.431	0.081	0.007	-0.590	-0.272	-5.302	0.000	164	1.62
Loughnan et al., 2019a	Combined	-0.736	0.157	0.025	-1.045	-0.427	-4.674	0.000	41	1.41
Loughnan et al., 2019b	Combined	-0.759	0.137	0.019	-1.028	-0.491	-5.539	0.000	55	1.48
Lukas et al., 2021	Combined	-0.169	0.147	0.022	-0.457	0.119	-1.151	0.250	37	1.45
McCloud et al., 2020	HADS	-0.407	0.102	0.010	-0.607	-0.207	-3.994	0.000	84	1.57
Meglic et al., 2010	BDI-II	-1.225	0.361	0.130	-1.932	-0.518	-3.396	0.001	10	0.82
Meyer et al., 2015	PHQ-9	-0.643	0.107	0.011	-0.852	-0.433	-6.001	0.000	85	1.56
Milgrom et al., 2016	BDI-II	-0.392	0.194	0.037	-0.771	-0.012	-2.023	0.043	22	1.30
Mira et al., 2017	Combined	-0.050	0.134	0.018	-0.313	0.214	-0.369	0.712	44	1.48
Montero-Marin et al., 2016	BDI-II	-0.107	0.097	0.009	-0.297	0.083	-1.103	0.270	86	1.58
Newby et al., 2013	Combined	-0.181	0.124	0.015	-0.424	0.061	-1.463	0.143	53	1.51
Noguchi et al., 2017	Combined	-0.307	0.051	0.003	-0.407	-0.206	-5.985	0.000	325	1.67
O'Mahen et al., 2014	EPDS	-1.427	0.197	0.039	-1.813	-1.042	-7.255	0.000	42	1.29
Oehler et al., 2020	Combined	-0.555	0.074	0.005	-0.699	-0.411	-7.541	0.000	174	1.63
Ofoegbu et al., 2020	BDI-II	0.218	0.093	0.009	0.036	0.399	2.345	0.019	96	1.59
Pfeiffer et al., 2020	QIRS-SR	-0.537	0.076	0.006	-0.685	-0.389	-7.108	0.000	163	1.63
Philips et al., 2014	PHQ-9	-0.672	0.056	0.003	-0.782	-0.562	-11.982	0.000	318	1.66
Pots et al., 2016a	CES-D	-1.212	0.145	0.021	-1.495	-0.928	-8.372	0.000	67	1.45
Pots et al., 2016b	CES-D	-0.893	0.114	0.013	-1.116	-0.669	-7.825	0.000	87	1.54
Proudfoot et al., 2003	BDI-II	-0.887	0.145	0.021	-1.172	-0.602	-6.104	0.000	53	1.45
Proudfoot et al., 2004	BDI-II	-0.804	0.096	0.009	-0.993	-0.615	-8.355	0.000	116	1.58
Proudfoot et al., 2013a	DASS-21-D	-0.271	0.059	0.003	-0.386	-0.156	-4.627	0.000	246	1.66
Reins et al., 2019	HRSD	-0.859	0.129	0.017	-1.113	-0.606	-6.646	0.000	66	1.50
Ritvo et al., 2021	Combined	-0.774	0.229	0.052	-1.222	-0.325	-3.383	0.001	23	1.19
Roepke et al., 2015	CES-D	-0.547	0.100	0.010	-0.743	-0.351	-5.471	0.000	93	1.58
Rollman et al., 2018	PROMIS	-0.862	0.105	0.011	-1.068	-0.657	-8.217	0.000	101	1.56
Rosso et al., 2016	Combined	-0.547	0.153	0.023	-0.845	-0.248	-3.583	0.000	40	1.43
Salisbury et al., 2016	PHQ-9	-0.512	0.055	0.003	-0.621	-0.404	-9.264	0.000	302	1.66
Sandoval et al., 2017	BDI-II	0.280	0.198	0.039	-0.109	0.669	1.410	0.158	20	1.29
Smith et al., 2017	PHQ-9	-0.582	0.126	0.016	-0.829	-0.335	-4.615	0.000	59	1.51
Sun et al., 2021	EPDS	-0.468	0.103	0.011	-0.670	-0.266	-4.533	0.000	84	1.57
Tönnies et al., 2021	PHQ-9	-0.739	0.192	0.037	-1.115	-0.362	-3.845	0.000	27	1.31
Tønning et al., 2021	Combined	-0.716	0.144	0.021	-0.998	-0.434	-4.975	0.000	50	1.46
Warmerdam et al., 2009	CES-D	-0.630	0.106	0.011	-0.837	-0.423	-5.973	0.000	87	1.56
Segal et al., 2020	PHQ-9	0.028	0.060	0.004	-0.089	0.144	0.465	0.642	230	1.66
Zwerenz et al., 2017	BDI-II	-0.599	0.093	0.009	-0.782	-0.416	-6.427	0.000	110	1.59
		-0.549	0.045	0.002	-0.638	-0.460	-12.103	0.000		

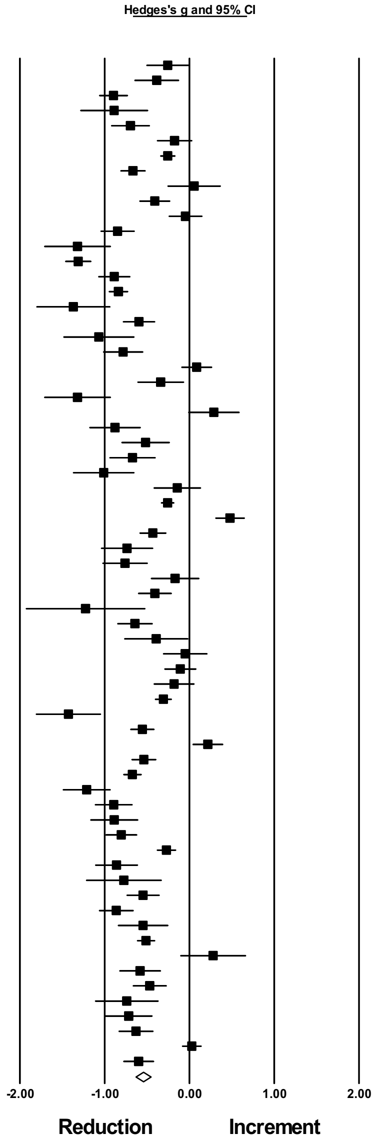

Meta Analysis

Multimedia Appendix 3.2.2
Forrest plot of short-term effects by control type

Short-term effects by control type

Group by Control_type	Study name	Outcome	Hedges's g	Standard error	Variance	Lower limit	Upper limit	Z-Value	p-Value	Total	Relative weight
			Statistics for each study								
Active	Andersson et al., 2005	Combined	-0.256	0.130	0.017	-0.510	-0.001	-1.970	0.049	49	11.21
Active	Berger et al., 2018	BDI-II	-0.385	0.135	0.018	-0.650	-0.121	-2.858	0.004	47	11.10
Active	Bohlmeijer et al., 2021a	CES-D	-0.696	0.117	0.014	-0.925	-0.466	-5.940	0.000	73	11.50
Active	Hoorelbeke & Koster, 2017	BDI-II	0.288	0.155	0.024	-0.016	0.592	1.859	0.063	34	10.61
Active	Johansson et al., 2012a	BDI-II	-0.878	0.155	0.024	-1.182	-0.573	-5.654	0.000	46	10.60
Active	Johansson et al., 2012b	Combined	-0.517	0.146	0.021	-0.804	-0.230	-3.532	0.000	42	10.82
Active	Johansson et al., 2013	PHQ-9	-0.671	0.140	0.020	-0.945	-0.397	-4.793	0.000	50	10.97
Active	Oehler et al., 2020	Combined	-0.555	0.074	0.005	-0.699	-0.411	-7.541	0.000	174	12.33
Active	Pots et al., 2016a	CES-D	-1.212	0.145	0.021	-1.495	-0.928	-8.372	0.000	67	10.86
Active			-0.543	0.119	0.014	-0.777	-0.310	-4.562	0.000		
CAU	Boschloo et al., 2019	PHQ-9	-0.256	0.046	0.002	-0.346	-0.166	-5.564	0.000	399	3.79
CAU	De Graaf et al., 2011	BDI-II	-0.848	0.103	0.011	-1.051	-0.645	-8.194	0.000	103	3.62
CAU	Eriksson et al., 2017	BDI-II	-1.320	0.201	0.041	-1.715	-0.925	-6.555	0.000	37	3.13
CAU	Gilbody et al., 2015	PHQ-9	-1.312	0.080	0.006	-1.468	-1.155	-16.446	0.000	239	3.70
CAU	Hallgren et al., 2016	MADRS-S	-0.837	0.059	0.004	-0.954	-0.721	-14.077	0.000	312	3.76
CAU	Hange et al., 2017	MADRS-S	-1.369	0.223	0.050	-1.807	-0.931	-6.127	0.000	31	3.00
CAU	Holst et al., 2018	BDI-II	-1.320	0.201	0.041	-1.715	-0.925	-6.555	0.000	37	3.13
CAU	Kivi et al., 2014	Combined	-1.011	0.185	0.034	-1.375	-0.648	-5.453	0.000	35	3.22
CAU	Kladnitski et al., 2020	PHQ-9	-0.143	0.143	0.020	-0.423	0.137	-1.001	0.317	39	3.44
CAU	Klein et al., 2017	Combined	-0.257	0.041	0.002	-0.338	-0.177	-6.282	0.000	504	3.80
CAU	Kok et al., 2015	IDS-SR	0.479	0.089	0.008	0.304	0.654	5.361	0.000	113	3.67
CAU	Loughnan et al., 2019a	Combined	-0.736	0.157	0.025	-1.045	-0.427	-4.674	0.000	41	3.37
CAU	Loughnan et al., 2019b	Combined	-0.759	0.137	0.019	-1.028	-0.491	-5.539	0.000	55	3.47
CAU	McCloud et al., 2020	HADS	-0.407	0.102	0.010	-0.607	-0.207	-3.994	0.000	84	3.63
CAU	Meglic et al., 2010	BDI-II	-1.225	0.361	0.130	-1.932	-0.518	-3.396	0.001	10	2.22
CAU	Meyer et al., 2015	PHQ-9	-0.643	0.107	0.011	-0.852	-0.433	-6.001	0.000	85	3.61
CAU	Montero-Marin et al., 2016	BDI-II	-0.107	0.097	0.009	-0.297	0.083	-1.103	0.270	86	3.64
CAU	O'Mahen et al., 2014	EPDS	-1.427	0.197	0.039	-1.813	-1.042	-7.255	0.000	42	3.15
CAU	Ofoegbu et al., 2020	BDI-II	0.218	0.093	0.009	0.036	0.399	2.345	0.019	96	3.66
CAU	Proudfoot et al., 2003	BDI-II	-0.887	0.145	0.021	-1.172	-0.602	-6.104	0.000	53	3.43
CAU	Proudfoot et al., 2004	BDI-II	-0.804	0.096	0.009	-0.993	-0.615	-8.355	0.000	116	3.65
CAU	Ritvo et al., 2021	Combined	-0.774	0.229	0.052	-1.222	-0.325	-3.383	0.001	23	2.97
CAU	Roepke et al., 2015	CES-D	-0.547	0.100	0.010	-0.743	-0.351	-5.471	0.000	93	3.63
CAU	Rollman et al., 2018	PROMIS	-0.862	0.105	0.011	-1.068	-0.657	-8.217	0.000	101	3.61
CAU	Salisbury et al., 2016	PHQ-9	-0.512	0.055	0.003	-0.621	-0.404	-9.264	0.000	302	3.77
CAU	Smith et al., 2017	PHQ-9	-0.582	0.126	0.016	-0.829	-0.335	-4.615	0.000	59	3.52
CAU	Tönnies et al., 2021	PHQ-9	-0.739	0.192	0.037	-1.115	-0.362	-3.845	0.000	27	3.18
CAU	Tønning et al., 2021	Combined	-0.716	0.144	0.021	-0.998	-0.434	-4.975	0.000	50	3.44
CAU	Segal et al., 2020	PHQ-9	0.028	0.060	0.004	-0.089	0.144	0.465	0.642	230	3.76
CAU			-0.652	0.083	0.007	-0.814	-0.489	-7.861	0.000		
Information	Birney et al., 2016	PHQ-9	-0.895	0.087	0.008	-1.066	-0.724	-10.269	0.000	150	9.81
Information	Braun et al., 2021	QIDS-SR	-0.666	0.077	0.006	-0.816	-0.516	-8.681	0.000	169	10.04
Information	Clarke et al., 2005	CES-D	-0.409	0.094	0.009	-0.592	-0.225	-4.365	0.000	100	9.67
Information	Clarke et al., 2009	PHQ-8	-0.046	0.102	0.010	-0.247	0.154	-0.454	0.650	77	9.46
Information	Harrer et al., 2021	CES-D	-0.595	0.098	0.010	-0.786	-0.404	-6.095	0.000	100	9.57
Information	Hatcher et al., 2018	PHQ-9	-1.069	0.214	0.046	-1.489	-0.649	-4.985	0.000	27	6.56
Information	Heim et al., 2021	PHQ-9	-0.782	0.122	0.015	-1.021	-0.543	-6.422	0.000	71	8.97
Information	Milgrom et al., 2016	BDI-II	-0.392	0.194	0.037	-0.771	-0.012	-2.023	0.043	22	7.08
Information	Proudfoot et al., 2013a	DASS-21-D	-0.271	0.059	0.003	-0.386	-0.156	-4.627	0.000	246	10.37
Information	Reins et al., 2019	HRSD	-0.859	0.129	0.017	-1.113	-0.606	-6.646	0.000	66	8.78
Information	Zwerenz et al., 2017	BDI-II	-0.599	0.093	0.009	-0.782	-0.416	-6.427	0.000	110	9.68
Information			-0.585	0.087	0.008	-0.756	-0.414	-6.711	0.000		
Monitoring	Tulbure et al., 2018	BDI-II	-0.888	0.205	0.042	-1.290	-0.486	-4.328	0.000	26	15.59
Monitoring	Holländare et al., 2011	Combined	-0.339	0.141	0.020	-0.615	-0.062	-2.399	0.016	42	25.70
Monitoring	Rosso et al., 2016	Combined	-0.547	0.153	0.023	-0.845	-0.248	-3.583	0.000	40	23.42
Monitoring	Sun et al., 2021	EPDS	-0.468	0.103	0.011	-0.670	-0.266	-4.533	0.000	84	35.29
Monitoring			-0.519	0.094	0.009	-0.702	-0.335	-5.535	0.000		
Sham	Pfeiffer et al., 2020	QIRS-SR	-0.537	0.076	0.006	-0.685	-0.389	-7.108	0.000	163	43.00
Sham	Philips et al., 2014	PHQ-9	-0.672	0.056	0.003	-0.782	-0.562	-11.982	0.000	318	57.00
Sham			-0.614	0.067	0.004	-0.746	-0.483	-9.171	0.000		
WL	Bohlmeijer et al., 2021b	CES-D	-0.175	0.107	0.011	-0.385	0.035	-1.637	0.102	71	8.57
WL	Choi et al., 2012	BDI-II	0.055	0.161	0.026	-0.260	0.371	0.344	0.731	30	7.54
WL	Hallford et al., 2021	PHQ-9	-0.886	0.097	0.009	-1.077	-0.696	-9.126	0.000	120	8.74
WL	Hobfoll et al., 2016	CESD-10	0.086	0.093	0.009	-0.096	0.268	0.926	0.355	94	8.81
WL	Lokman et al., 2017	IDS-SR	-0.431	0.081	0.007	-0.590	-0.272	-5.302	0.000	164	8.98
WL	Lukas et al., 2021	Combined	-0.169	0.147	0.022	-0.457	0.119	-1.151	0.250	37	7.83
WL	Mira et al., 2017	Combined	-0.050	0.134	0.018	-0.313	0.214	-0.369	0.712	44	8.07
WL	Newby et al., 2013	Combined	-0.181	0.124	0.015	-0.424	0.061	-1.463	0.143	53	8.27
WL	Noguchi et al., 2017	Combined	-0.307	0.051	0.003	-0.407	-0.206	-5.985	0.000	325	9.35
WL	Pots et al., 2016b	CES-D	-0.893	0.114	0.013	-1.116	-0.669	-7.825	0.000	87	8.45
WL	Sandoval et al., 2017	BDI-II	0.280	0.198	0.039	-0.109	0.669	1.410	0.158	20	6.79
WL	Warmerdam et al., 2009	CES-D	-0.630	0.106	0.011	-0.837	-0.423	-5.973	0.000	87	8.60
WL			-0.291	0.095	0.009	-0.478	-0.104	-3.048	0.002		
Overall			-0.552	0.035	0.001	-0.621	-0.482	-15.544	0.000		

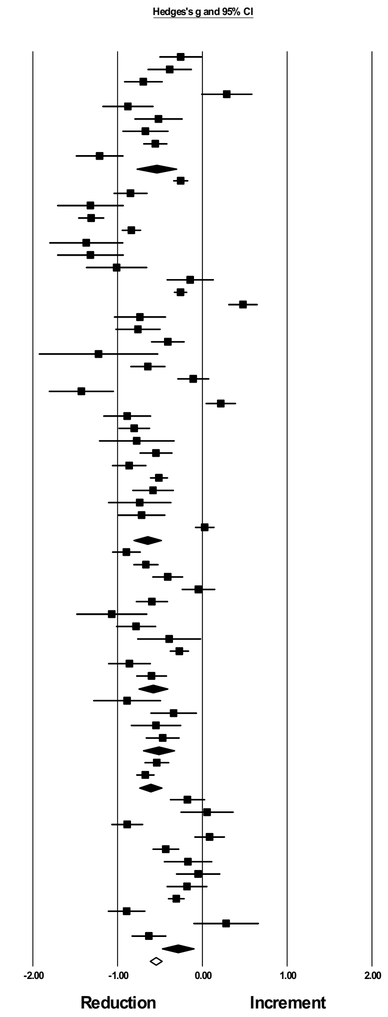

Meta Analysis

Multimedia Appendix 3.2.3
Forrest plot of short-term effects by severity

Short-term effects by severity

Group by Severity	Studyname	Outcome	Hedges's g	Standard error	Variance	Lower limit	Upper limit	Z-Value	p-Value	Total	Relative weight
			Statistics for each study								
Mild	Bohlmeijer et al., 2021a	CES-D	-0.696	0.117	0.014	-0.925	-0.466	-5.940	0.000	73	9.84
Mild	Bohlmeijer et al., 2021b	CES-D	-0.175	0.107	0.011	-0.385	0.035	-1.637	0.102	71	9.98
Mild	Braun et al., 2021	QIDS-SR	-0.666	0.077	0.006	-0.816	-0.516	-8.681	0.000	169	10.35
Mild	Holländare et al., 2011	Combined	-0.339	0.141	0.020	-0.615	-0.062	-2.399	0.016	42	9.47
Mild	Hoorelbeke & Koster, 2017	BDI-II	0.288	0.155	0.024	-0.016	0.592	1.859	0.063	34	9.24
Mild	Kok et al., 2015	IDS-SR	0.479	0.089	0.008	0.304	0.654	5.361	0.000	113	10.21
Mild	Lokman et al., 2017	IDS-SR	-0.431	0.081	0.007	-0.590	-0.272	-5.302	0.000	164	10.30
Mild	McCloud et al., 2020	HADS	-0.407	0.102	0.010	-0.607	-0.207	-3.994	0.000	84	10.05
Mild	Sun et al., 2021	EPDS	-0.468	0.103	0.011	-0.670	-0.266	-4.533	0.000	84	10.03
Mild	Segal et al., 2020	PHQ-9	0.028	0.060	0.004	-0.089	0.144	0.465	0.642	230	10.51
Mild			-0.241	0.125	0.016	-0.486	0.004	-1.926	0.054		
Moderate	Andersson et al., 2005	Combined	-0.256	0.130	0.017	-0.510	-0.001	-1.970	0.049	49	3.02
Moderate	Birney et al., 2016	PHQ-9	-0.895	0.087	0.008	-1.066	-0.724	-10.269	0.000	150	3.31
Moderate	Boschloo et al., 2019	PHQ-9	-0.256	0.046	0.002	-0.346	-0.166	-5.564	0.000	399	3.52
Moderate	Choi et al., 2012	BDI-II	0.055	0.161	0.026	-0.260	0.371	0.344	0.731	30	2.77
Moderate	Clarke et al., 2009	PHQ-8	-0.046	0.102	0.010	-0.247	0.154	-0.454	0.650	77	3.22
Moderate	De Graaf et al., 2011	BDI-II	-0.848	0.103	0.011	-1.051	-0.645	-8.194	0.000	103	3.21
Moderate	Eriksson et al., 2017	BDI-II	-1.320	0.201	0.041	-1.715	-0.925	-6.555	0.000	37	2.46
Moderate	Hallgren et al., 2016	MADRS-S	-0.837	0.059	0.004	-0.954	-0.721	-14.077	0.000	312	3.46
Moderate	Hange et al., 2017	MADRS-S	-1.369	0.223	0.050	-1.807	-0.931	-6.127	0.000	31	2.29
Moderate	Harrer et al., 2021	CES-D	-0.595	0.098	0.010	-0.786	-0.404	-6.095	0.000	100	3.25
Moderate	Holst et al., 2018	BDI-II	-1.320	0.201	0.041	-1.715	-0.925	-6.555	0.000	37	2.46
Moderate	Johansson et al., 2012a	BDI-II	-0.878	0.155	0.024	-1.182	-0.573	-5.654	0.000	46	2.82
Moderate	Johansson et al., 2012b	Combined	-0.517	0.146	0.021	-0.804	-0.230	-3.532	0.000	42	2.89
Moderate	Johansson et al., 2013	PHQ-9	-0.671	0.140	0.020	-0.945	-0.397	-4.793	0.000	50	2.94
Moderate	Kivi et al., 2014	Combined	-1.011	0.185	0.034	-1.375	-0.648	-5.453	0.000	35	2.58
Moderate	Kladnitski et al., 2020	PHQ-9	-0.143	0.143	0.020	-0.423	0.137	-1.001	0.317	39	2.92
Moderate	Loughnan et al., 2019a	Combined	-0.736	0.157	0.025	-1.045	-0.427	-4.674	0.000	41	2.80
Moderate	Loughnan et al., 2019b	Combined	-0.759	0.137	0.019	-1.028	-0.491	-5.539	0.000	55	2.96
Moderate	Meglic et al., 2010	BDI-II	-1.225	0.361	0.130	-1.932	-0.518	-3.396	0.001	10	1.44
Moderate	Milgrom et al., 2016	BDI-II	-0.392	0.194	0.037	-0.771	-0.012	-2.023	0.043	22	2.52
Moderate	Montero-Marin et al., 2016	BDI-II	-0.107	0.097	0.009	-0.297	0.083	-1.103	0.270	86	3.25
Moderate	Newby et al., 2013	Combined	-0.181	0.124	0.015	-0.424	0.061	-1.463	0.143	53	3.06
Moderate	Noguchi et al., 2017	Combined	-0.307	0.051	0.003	-0.407	-0.206	-5.985	0.000	325	3.50
Moderate	Oehler et al., 2020	Combined	-0.555	0.074	0.005	-0.699	-0.411	-7.541	0.000	174	3.39
Moderate	Pfeiffer et al., 2020	QIRS-SR	-0.537	0.076	0.006	-0.685	-0.389	-7.108	0.000	163	3.38
Moderate	Philips et al., 2014	PHQ-9	-0.672	0.056	0.003	-0.782	-0.562	-11.982	0.000	318	3.48
Moderate	Proudfoot et al., 2003	BDI-II	-0.887	0.145	0.021	-1.172	-0.602	-6.104	0.000	53	2.90
Moderate	Proudfoot et al., 2004	BDI-II	-0.804	0.096	0.009	-0.993	-0.615	-8.355	0.000	116	3.26
Moderate	Proudfoot et al., 2013a	DASS-21-D	-0.271	0.059	0.003	-0.386	-0.156	-4.627	0.000	246	3.47
Moderate	Reins et al., 2019	HRSD	-0.859	0.129	0.017	-1.113	-0.606	-6.646	0.000	66	3.02
Moderate	Ritvo et al., 2021	Combined	-0.774	0.229	0.052	-1.222	-0.325	-3.383	0.001	23	2.25
Moderate	Rollman et al., 2018	PROMIS	-0.862	0.105	0.011	-1.068	-0.657	-8.217	0.000	101	3.20
Moderate	Sandoval et al., 2017	BDI-II	0.280	0.198	0.039	-0.109	0.669	1.410	0.158	20	2.48
Moderate	Tönnies et al., 2021	PHQ-9	-0.739	0.192	0.037	-1.115	-0.362	-3.845	0.000	27	2.53
Moderate			-0.602	0.056	0.003	-0.712	-0.493	-10.782	0.000		
Severe	Berger et al., 2018	BDI-II	-0.385	0.135	0.018	-0.650	-0.121	-2.858	0.004	47	5.49
Severe	Tulbure et al., 2018	BDI-II	-0.888	0.205	0.042	-1.290	-0.486	-4.328	0.000	26	4.79
Severe	Clarke et al., 2005	CES-D	-0.409	0.094	0.009	-0.592	-0.225	-4.365	0.000	100	5.83
Severe	Gilbody et al., 2015	PHQ-9	-1.312	0.080	0.006	-1.468	-1.155	-16.446	0.000	239	5.92
Severe	Hallford et al., 2021	PHQ-9	-0.886	0.097	0.009	-1.077	-0.696	-9.126	0.000	120	5.80
Severe	Hatcher et al., 2018	PHQ-9	-1.069	0.214	0.046	-1.489	-0.649	-4.985	0.000	27	4.69
Severe	Heim et al., 2021	PHQ-9	-0.782	0.122	0.015	-1.021	-0.543	-6.422	0.000	71	5.61
Severe	Lukas et al., 2021	Combined	-0.169	0.147	0.022	-0.457	0.119	-1.151	0.250	37	5.38
Severe	Meyer et al., 2015	PHQ-9	-0.643	0.107	0.011	-0.852	-0.433	-6.001	0.000	85	5.73
Severe	O'Mahen et al., 2014	EPDS	-1.427	0.197	0.039	-1.813	-1.042	-7.255	0.000	42	4.87
Severe	Ofoegbu et al., 2020	BDI-II	0.218	0.093	0.009	0.036	0.399	2.345	0.019	96	5.84
Severe	Pots et al., 2016a	CES-D	-1.212	0.145	0.021	-1.495	-0.928	-8.372	0.000	67	5.40
Severe	Pots et al., 2016b	CES-D	-0.893	0.114	0.013	-1.116	-0.669	-7.825	0.000	87	5.67
Severe	Roepke et al., 2015	CES-D	-0.547	0.100	0.010	-0.743	-0.351	-5.471	0.000	93	5.78
Severe	Salisbury et al., 2016	PHQ-9	-0.512	0.055	0.003	-0.621	-0.404	-9.264	0.000	302	6.06
Severe	Smith et al., 2017	PHQ-9	-0.582	0.126	0.016	-0.829	-0.335	-4.615	0.000	59	5.57
Severe	Warmerdam et al., 2009	CES-D	-0.630	0.106	0.011	-0.837	-0.423	-5.973	0.000	87	5.74
Severe	Zwerenz et al., 2017	BDI-II	-0.599	0.093	0.009	-0.782	-0.416	-6.427	0.000	110	5.83
Severe			-0.696	0.094	0.009	-0.880	-0.511	-7.373	0.000		
Overall			-0.577	0.045	0.002	-0.665	-0.489	-12.857	0.000		

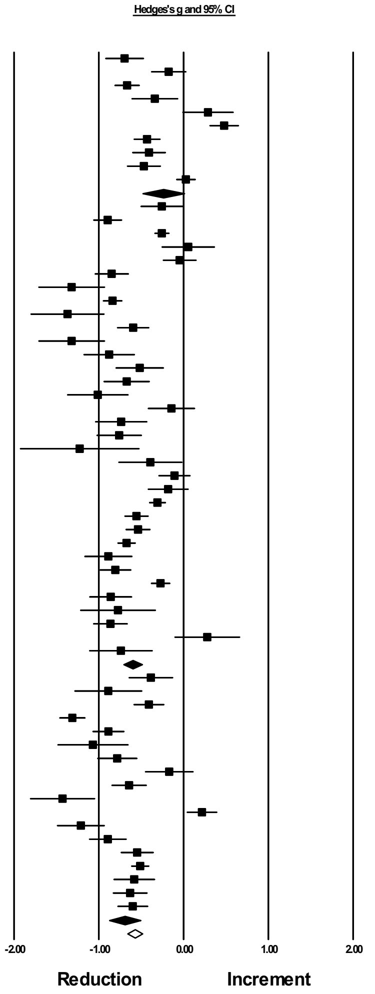

Meta Analysis

Multimedia Appendix 3.3.1
Forrest plot of medium-term effects

Medium-term effects

Study name	Outcome	Hedges's g	Standard error	Variance	Lower limit	Upper limit	Z-Value	p-Value	Total	Relative weight
Andersson et al., 2005	Combined	-0.885	0.151	0.023	-1.181	-0.589	-5.860	0.000	49	3.94
Berger et al., 2018	BDI-II	-0.592	0.141	0.020	-0.869	-0.315	-4.193	0.000	47	4.03
Bohlmeijer et al., 2021a	CES-D	-0.380	0.109	0.012	-0.593	-0.167	-3.501	0.000	73	4.30
Buntrock et al., 2015	CES-D	-0.701	0.071	0.005	-0.839	-0.562	-9.932	0.000	204	4.57
Clarke et al., 2005	CES-D	-0.413	0.094	0.009	-0.596	-0.229	-4.407	0.000	100	4.42
Clarke et al., 2009	PHQ-8	-0.090	0.102	0.010	-0.290	0.111	-0.877	0.381	77	4.35
De Graaf et al., 2011	BDI-II	-1.037	0.110	0.012	-1.253	-0.821	-9.419	0.000	103	4.29
Flygare et al., 2020	Combined	-1.086	0.165	0.027	-1.410	-0.763	-6.588	0.000	47	3.81
Geraedts et al., 2014	CES-D	-1.411	0.119	0.014	-1.644	-1.178	-11.868	0.000	115	4.22
Gilbody et al., 2015	PHQ-9	-1.107	0.074	0.006	-1.252	-0.961	-14.911	0.000	239	4.55
Hange et al., 2017	MADRS-S	-1.403	0.226	0.051	-1.846	-0.959	-6.202	0.000	31	3.23
Hoifodt et al., 2013	BDI-II	-0.951	0.147	0.022	-1.239	-0.663	-6.468	0.000	54	3.98
Holländare et al., 2011	Combined	-0.611	0.150	0.022	-0.905	-0.317	-4.075	0.000	42	3.95
Kessler et al., 2009	BDI-II	-0.829	0.086	0.007	-0.998	-0.661	-9.647	0.000	148	4.47
Klein et al., 2017	PHQ-9	-0.296	0.041	0.002	-0.377	-0.215	-7.194	0.000	504	4.71
Levesque et al., 2011	BDI-II	-0.585	0.074	0.005	-0.730	-0.441	-7.954	0.000	176	4.55
Levin et al., 2011	CES-D	-0.583	0.102	0.010	-0.783	-0.383	-5.722	0.000	91	4.36
Oehler et al., 2020	Combined	-0.772	0.078	0.006	-0.925	-0.619	-9.902	0.000	174	4.52
Proudfoot et al., 2003	BDI-II	-0.665	0.136	0.018	-0.931	-0.399	-4.897	0.000	53	4.08
Salisbury et al., 2016	PHQ-9	-0.826	0.060	0.004	-0.944	-0.708	-13.713	0.000	302	4.62
Eriksson et al., 2017	BDI-II	-1.498	0.215	0.046	-1.918	-1.077	-6.975	0.000	37	3.34
Gili et al., 2020	PHQ-9	-0.751	0.134	0.018	-1.015	-0.488	-5.591	0.000	57	4.09
Holst et al., 2018	BDI-II	-1.498	0.215	0.046	-1.918	-1.077	-6.975	0.000	37	3.34
Rollman et al., 2018	PROMIS	-1.027	0.111	0.012	-1.244	-0.810	-9.268	0.000	101	4.29
		-0.810	0.071	0.005	-0.950	-0.670	-11.344	0.000		

Hedges's g and 95% CI

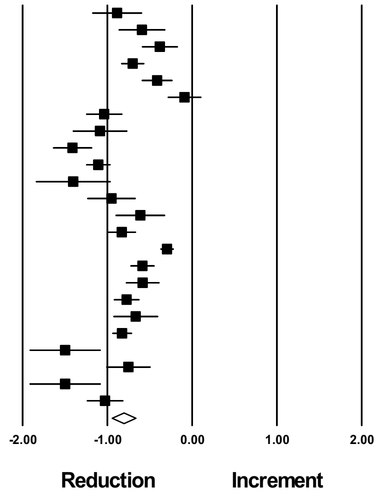

Meta Analysis

Multimedia Appendix 3.3.2
Forrest plot of medium-term effects by control type

Medium-term effects by control type

Group by Control_type	Studyname	Outcome	Hedges's g	Standard error	Variance	Lower limit	Upper limit	Z-Value	p-Value	Total	Relative weight
			Statistics for each study								
Active	Andersson et al., 2005	Combined	-0.885	0.151	0.023	-1.181	-0.589	-5.860	0.000	49	21.33
Active	Berger et al., 2018	BDI-II	-0.592	0.141	0.020	-0.869	-0.315	-4.193	0.000	47	22.43
Active	Bohlmeijer et al., 2021a	CES-D	-0.380	0.109	0.012	-0.593	-0.167	-3.501	0.000	73	26.30
Active	Oehler et al., 2020	Combined	-0.772	0.078	0.006	-0.925	-0.619	-9.902	0.000	174	29.94
Active			-0.653	0.111	0.012	-0.871	-0.435	-5.865	0.000		
CAU	De Graaf et al., 2011	BDI-II	-1.037	0.110	0.012	-1.253	-0.821	-9.419	0.000	103	7.39
CAU	Eriksson et al., 2017	BDI-II	-1.498	0.215	0.046	-1.918	-1.077	-6.975	0.000	37	6.08
CAU	Geraedts et al., 2014	CES-D	-1.411	0.119	0.014	-1.644	-1.178	-11.868	0.000	115	7.30
CAU	Gilbody et al., 2015	PHQ-9	-1.107	0.074	0.006	-1.252	-0.961	-14.911	0.000	239	7.72
CAU	Gili et al., 2020	PHQ-9	-0.751	0.134	0.018	-1.015	-0.488	-5.591	0.000	57	7.12
CAU	Hange et al., 2017	MADRS-S	-1.403	0.226	0.051	-1.846	-0.959	-6.202	0.000	31	5.92
CAU	Hoifodt et al., 2013	BDI-II	-0.951	0.147	0.022	-1.239	-0.663	-6.468	0.000	54	6.97
CAU	Holst et al., 2018	BDI-II	-1.498	0.215	0.046	-1.918	-1.077	-6.975	0.000	37	6.08
CAU	Klein et al., 2017	PHQ-9	-0.296	0.041	0.002	-0.377	-0.215	-7.194	0.000	504	7.92
CAU	Levesque et al., 2011	BDI-II	-0.585	0.074	0.005	-0.730	-0.441	-7.954	0.000	176	7.72
CAU	Levin et al., 2011	CES-D	-0.583	0.102	0.010	-0.783	-0.383	-5.722	0.000	91	7.47
CAU	Proudfoot et al., 2003	BDI-II	-0.665	0.136	0.018	-0.931	-0.399	-4.897	0.000	53	7.11
CAU	Rollman et al., 2018	PROMIS	-1.027	0.111	0.012	-1.244	-0.810	-9.268	0.000	101	7.38
CAU	Salisbury et al., 2016	PHQ-9	-0.826	0.060	0.004	-0.944	-0.708	-13.713	0.000	302	7.81
CAU			-0.950	0.108	0.012	-1.161	-0.739	-8.807	0.000		
Information	Buntrock et al., 2015	CES-D	-0.701	0.071	0.005	-0.839	-0.562	-9.932	0.000	204	34.42
Information	Clarke et al., 2005	CES-D	-0.413	0.094	0.009	-0.596	-0.229	-4.407	0.000	100	33.08
Information	Clarke et al., 2009	PHQ-8	-0.090	0.102	0.010	-0.290	0.111	-0.877	0.381	77	32.50
Information			-0.407	0.179	0.032	-0.758	-0.055	-2.269	0.023		
Overall			-0.743	0.071	0.005	-0.883	-0.604	-10.452	0.000		

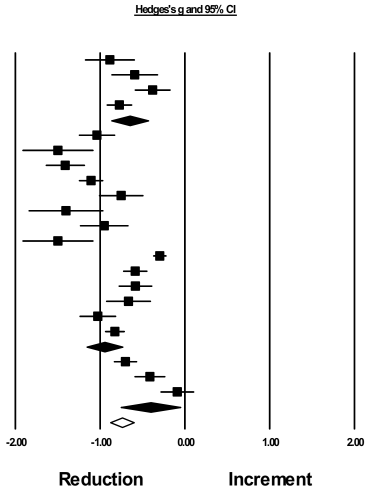

Meta Analysis

Multimedia Appendix 3.3.3
Forrest plot of medium-term effects by severity

Medium-term effects by severity

Group by Severity	Studyname	Outcome	Statistics for each study								
			Hedges's g	Standard error	Variance	Lower limit	Upper limit	Z-Value	p-Value	Total	Relative weight
Mild	Bohlmeijer et al., 2021a	CES-D	-0.380	0.109	0.012	-0.593	-0.167	-3.501	0.000	73	60.04
Mild	Holländare et al., 2011	Combined	-0.611	0.150	0.022	-0.905	-0.317	-4.075	0.000	42	39.96
Mild			-0.473	0.113	0.013	-0.694	-0.251	-4.186	0.000		
Moderate	Andersson et al., 2005	Combined	-0.885	0.151	0.023	-1.181	-0.589	-5.860	0.000	49	7.64
Moderate	Clarke et al., 2009	PHQ-8	-0.090	0.102	0.010	-0.290	0.111	-0.877	0.381	77	8.16
Moderate	De Graaf et al., 2011	BDI-II	-1.037	0.110	0.012	-1.253	-0.821	-9.419	0.000	103	8.09
Moderate	Eriksson et al., 2017	BDI-II	-1.498	0.215	0.046	-1.918	-1.077	-6.975	0.000	37	6.81
Moderate	Flygare et al., 2020	Combined	-1.086	0.165	0.027	-1.410	-0.763	-6.588	0.000	47	7.47
Moderate	Gili et al., 2020	PHQ-9	-0.751	0.134	0.018	-1.015	-0.488	-5.591	0.000	57	7.83
Moderate	Hange et al., 2017	MADRS-S	-1.403	0.226	0.051	-1.846	-0.959	-6.202	0.000	31	6.66
Moderate	Hoifodt et al., 2013	BDI-II	-0.951	0.147	0.022	-1.239	-0.663	-6.468	0.000	54	7.69
Moderate	Holst et al., 2018	BDI-II	-1.498	0.215	0.046	-1.918	-1.077	-6.975	0.000	37	6.81
Moderate	Klein et al., 2017	PHQ-9	-0.296	0.041	0.002	-0.377	-0.215	-7.194	0.000	504	8.58
Moderate	Oehler et al., 2020	Combined	-0.772	0.078	0.006	-0.925	-0.619	-9.902	0.000	174	8.37
Moderate	Proudfoot et al., 2003	BDI-II	-0.665	0.136	0.018	-0.931	-0.399	-4.897	0.000	53	7.82
Moderate	Rollman et al., 2018	PROMIS	-1.027	0.111	0.012	-1.244	-0.810	-9.268	0.000	101	8.08
Moderate			-0.894	0.121	0.015	-1.132	-0.657	-7.377	0.000		
Severe	Berger et al., 2018	BDI-II	-0.592	0.141	0.020	-0.869	-0.315	-4.193	0.000	47	10.91
Severe	Buntrock et al., 2015	CES-D	-0.701	0.071	0.005	-0.839	-0.562	-9.932	0.000	204	13.21
Severe	Clarke et al., 2005	CES-D	-0.413	0.094	0.009	-0.596	-0.229	-4.407	0.000	100	12.54
Severe	Geraedts et al., 2014	CES-D	-1.411	0.119	0.014	-1.644	-1.178	-11.868	0.000	115	11.70
Severe	Gilbody et al., 2015	PHQ-9	-1.107	0.074	0.006	-1.252	-0.961	-14.911	0.000	239	13.12
Severe	Kessler et al., 2009	BDI-II	-0.829	0.086	0.007	-0.998	-0.661	-9.647	0.000	148	12.78
Severe	Levin et al., 2011	CES-D	-0.583	0.102	0.010	-0.783	-0.383	-5.722	0.000	91	12.27
Severe	Salisbury et al., 2016	PHQ-9	-0.826	0.060	0.004	-0.944	-0.708	-13.713	0.000	302	13.47
Severe			-0.808	0.097	0.009	-0.997	-0.619	-8.359	0.000		
Overall			-0.727	0.063	0.004	-0.850	-0.604	-11.582	0.000		

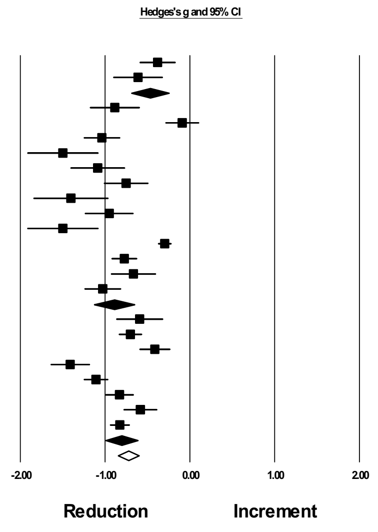

Meta Analysis

Multimedia Appendix 3.4.1 Forrest plot of long-term effects

Long-term effects

Study name	Outcome	Hedges's g	Standard error	Variance	Lower limit	Upper limit	Z-Value	p-Value	Total	Relative weight
		Statistics for each study								
Clarke et al., 2009	PHQ-8	-0.180	0.103	0.011	-0.381	0.022	-1.743	0.081	77	11.26
Flygare et al., 2020	Combined	-0.976	0.159	0.025	-1.287	-0.665	-6.150	0.000	47	10.37
Geraedts et al., 2014	CES-D	-1.028	0.104	0.011	-1.231	-0.824	-9.887	0.000	115	11.24
Gilbody et al., 2015	PHQ-9	-1.124	0.075	0.006	-1.270	-0.977	-15.050	0.000	239	11.60
Hallgren et al., 2016	MADRS-S	-1.197	0.067	0.004	-1.328	-1.065	-17.844	0.000	312	11.67
Hange et al., 2017	MADRS-S	-1.492	0.234	0.055	-1.950	-1.035	-6.387	0.000	31	8.95
Klein et al., 2017	Combined	-0.495	0.043	0.002	-0.578	-0.411	-11.594	0.000	504	11.87
Montero-Marin et al., 2016	BDI-II	-0.107	0.097	0.009	-0.297	0.083	-1.103	0.270	86	11.33
Zindel et al., 2020	PHQ-9	-0.494	0.063	0.004	-0.618	-0.371	-7.838	0.000	230	11.71
		-0.769	0.139	0.019	-1.041	-0.498	-5.551	0.000		

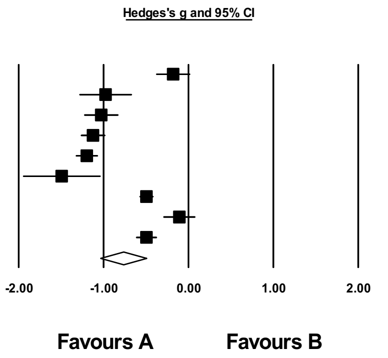

Meta Analysis

Multimedia Appendix 3.4.2 Forrest plot of long-term effects by control type

Long-term effects by control type

Group by Control_type	Study name		Hedges's g	Standard error	Variance	Lower limit	Upper limit	Z-Value	p-Value	Total	Relative weight
CAU	Geraedts et al., 2014	CES-D	-1.028	0.104	0.011	-1.231	-0.824	-9.887	0.000	115	14.34
CAU	Gilbody et al., 2015	PHQ-9	-1.124	0.075	0.006	-1.270	-0.977	-15.050	0.000	239	14.80
CAU	Hallgren et al., 2016	MADRS-S	-1.197	0.067	0.004	-1.328	-1.065	-17.844	0.000	312	14.90
CAU	Hange et al., 2017	MADRS-S	-1.492	0.234	0.055	-1.950	-1.035	-6.387	0.000	31	11.41
CAU	Klein et al., 2017	Combined	-0.495	0.043	0.002	-0.578	-0.411	-11.594	0.000	504	15.14
CAU	Montero-Marin et al., 2016	BDI-II	-0.107	0.097	0.009	-0.297	0.083	-1.103	0.270	86	14.46
CAU	Zindel et al., 2020	PHQ-9	-0.494	0.063	0.004	-0.618	-0.371	-7.838	0.000	230	14.94
CAU			-0.827	0.156	0.024	-1.133	-0.521	-5.293	0.000		
Overall			-0.827	0.156	0.024	-1.133	-0.521	-5.293	0.000		

Hedges's g and 95% CI

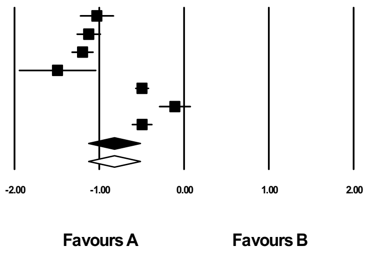

Meta Analysis

Multimedia Appendix 3.4.3 Forrest plot of long-term effects by severity

Long-term effects by severity

Group by Severity	Studyname	Outcome	Hedges's g	Standard error	Variance	Lower limit	Upper limit	Z-Value	p-Value	Total	Relative weight
			Statistics for each study								
Moderate	Clarke et al., 2009	PHQ-8	-0.180	0.103	0.011	-0.381	0.022	-1.743	0.081	77	20.48
Moderate	Flygare et al., 2020	Combined	-0.976	0.159	0.025	-1.287	-0.665	-6.150	0.000	47	19.75
Moderate	Hallgren et al., 2016	MADRS-S	-1.197	0.067	0.004	-1.328	-1.065	-17.844	0.000	312	20.80
Moderate	Hange et al., 2017	MADRS-S	-1.492	0.234	0.055	-1.950	-1.035	-6.387	0.000	31	18.42
Moderate	Montero-Marin et al., 2016	BDI-II	-0.107	0.097	0.009	-0.297	0.083	-1.103	0.270	86	20.54
Moderate			-0.775	0.284	0.081	-1.332	-0.219	-2.732	0.006		
Severe	Geraedts et al., 2014	CES-D	-1.028	0.104	0.011	-1.231	-0.824	-9.887	0.000	115	34.05
Severe	Gilbody et al., 2015	PHQ-9	-1.124	0.075	0.006	-1.270	-0.977	-15.050	0.000	239	65.95
Severe			-1.091	0.061	0.004	-1.210	-0.972	-17.992	0.000		
Overall			-1.077	0.059	0.004	-1.194	-0.961	-18.166	0.000		

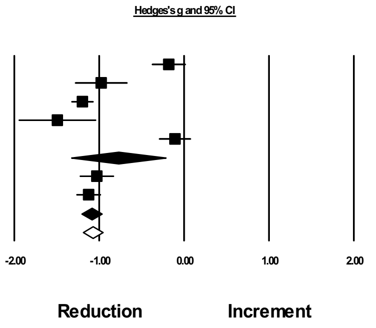

Meta Analysis
