# Supplementary figures and images for: Time-Dependent Changes in Depressive Symptoms Among Control Participants in Digital-Based Psychological Intervention Studies: Meta-analysis of Randomized Controlled Trials
Source: J Med Internet Res. 2023 Apr 12;25:e39029. doi: 10.2196/39029 (PMC10134030; doi:10.2196/39029)

Multimedia Appendix 4.1 – Funnel plot of immediate effects

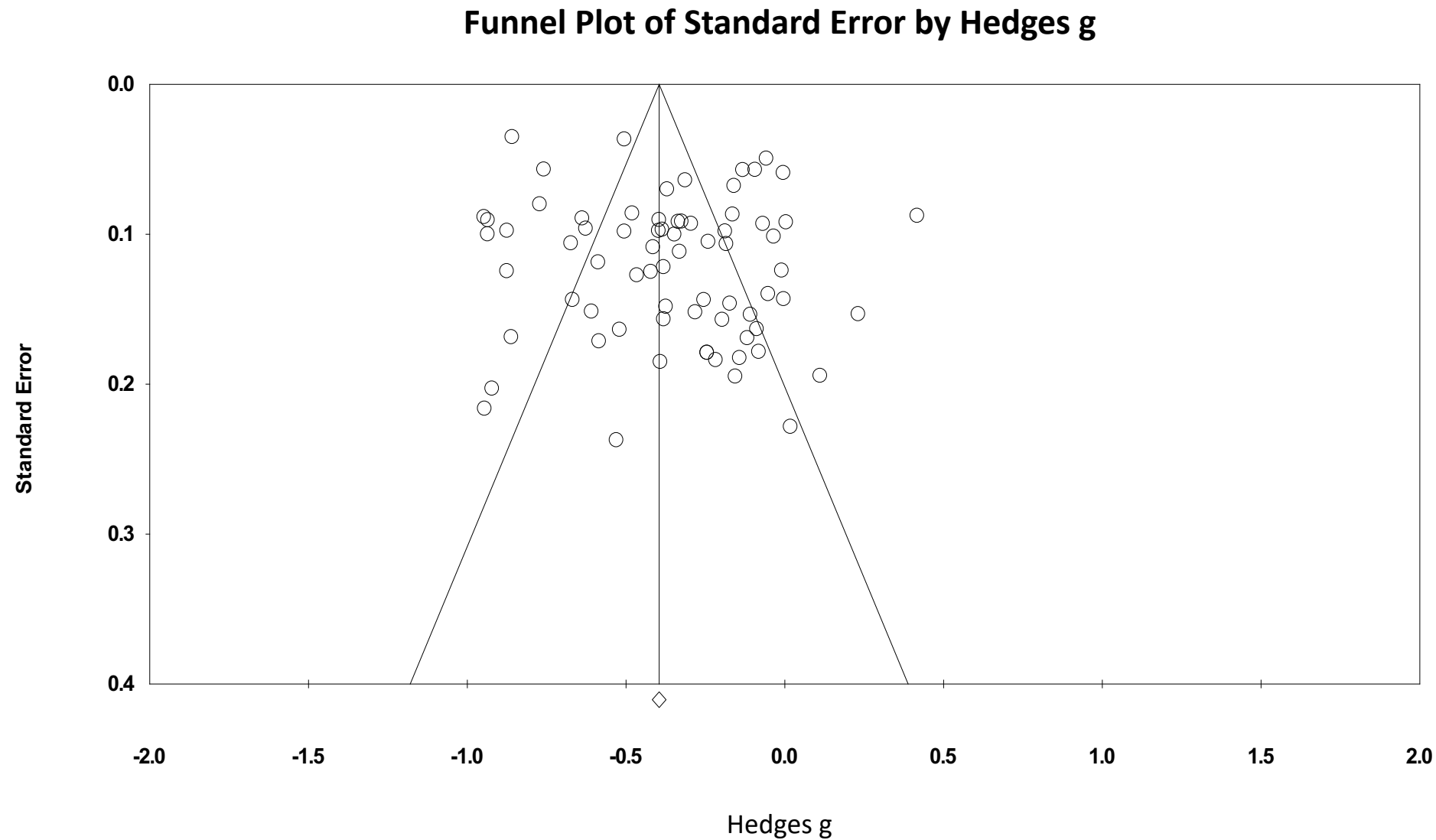

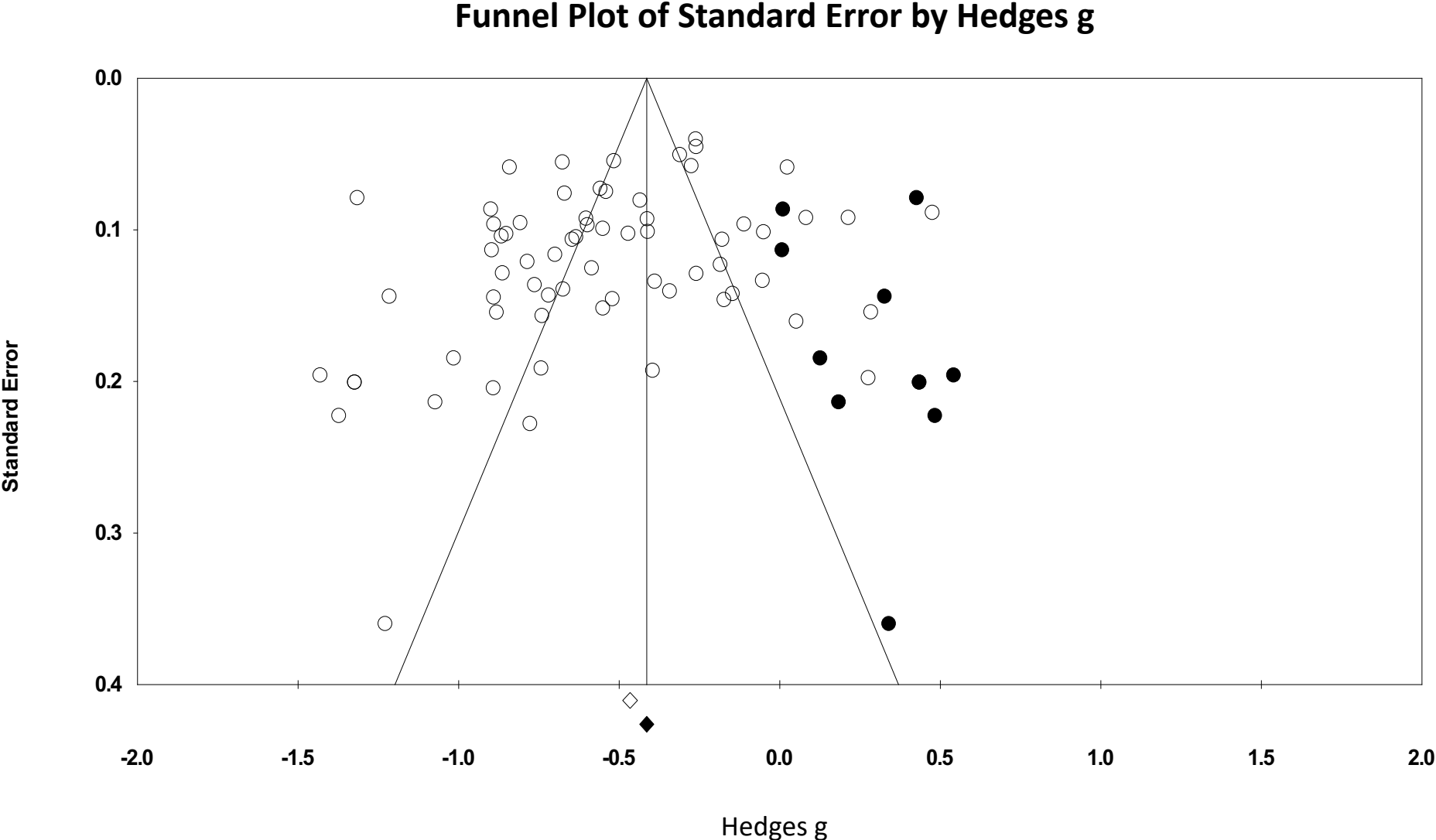

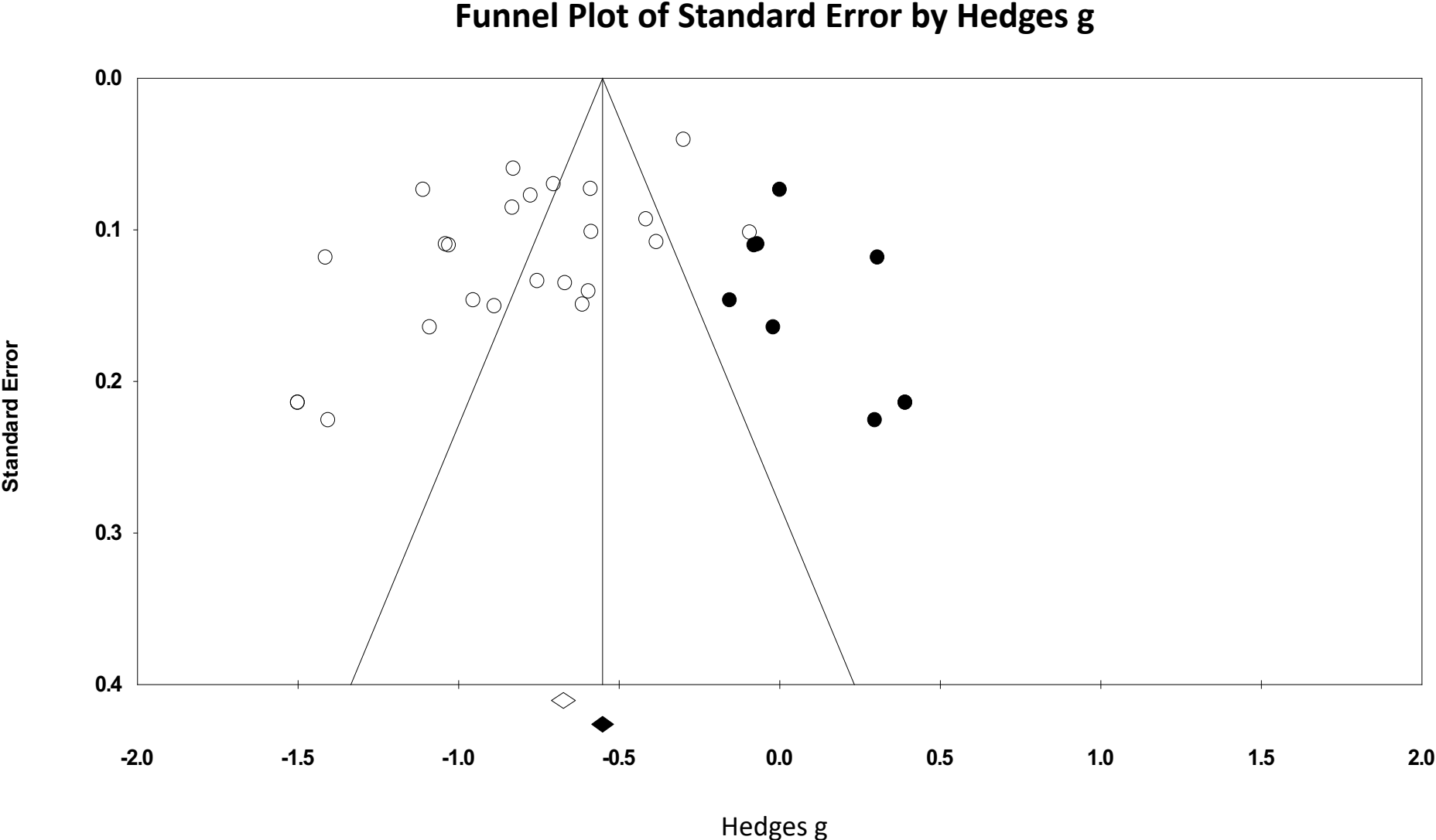

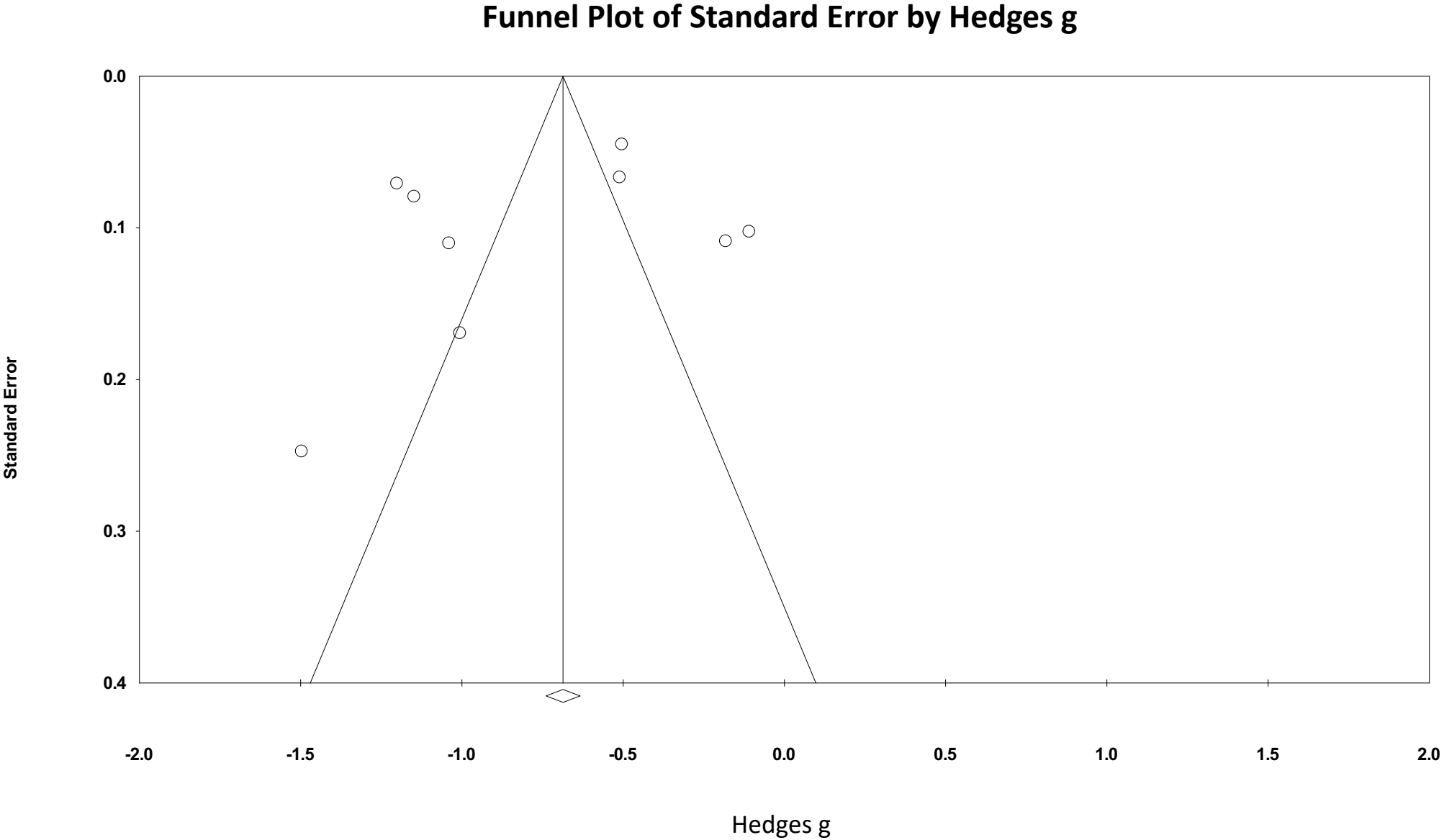

Supplement: Multimedia Appendix 4 [file jmir_v25i1e39029_app4.pdf]
